# A Foldamer-Dendrimer Conjugate Neutralizes Synaptotoxic β-Amyloid Oligomers

**DOI:** 10.1371/journal.pone.0039485

**Published:** 2012-07-30

**Authors:** Lívia Fülöp, István M. Mándity, Gábor Juhász, Viktor Szegedi, Anasztázia Hetényi, Edit Wéber, Zsolt Bozsó, Dóra Simon, Mária Benkő, Zoltán Király, Tamás A. Martinek

**Affiliations:** 1 Department of Medical Chemistry, University of Szeged, Szeged, Hungary; 2 Institute of Pharmaceutical Chemistry, University of Szeged, Szeged, Hungary; 3 Bay Zoltán Foundation for Applied Research – BAYGEN, Szeged, Hungary; 4 Department of Physical Chemistry and Materials Science, University of Szeged, Szeged, Hungary; Institute of Enzymology of the Hungarian Academy of Science, Hungary

## Abstract

**Background and Aims:**

Unnatural self-organizing biomimetic polymers (foldamers) emerged as promising materials for biomolecule recognition and inhibition. Our goal was to construct multivalent foldamer-dendrimer conjugates which wrap the synaptotoxic β-amyloid (Aβ) oligomers with high affinity through their helical foldamer tentacles. Oligomeric Aβ species play pivotal role in Alzheimer's disease, therefore recognition and direct inhibition of this undruggable target is a great current challenge.

**Methods and Results:**

Short helical β-peptide foldamers with designed secondary structures and side chain chemistry patterns were applied as potential recognition segments and their binding to the target was tested with NMR methods (saturation transfer difference and transferred-nuclear Overhauser effect). Helices exhibiting binding in the µM region were coupled to a tetravalent G0-PAMAM dendrimer. *In vitro* biophysical (isothermal titration calorimetry, dynamic light scattering, transmission electron microscopy and size-exclusion chromatography) and biochemical tests (ELISA and dot blot) indicated the tight binding between the foldamer conjugates and the Aβ oligomers. Moreover, a selective low nM interaction with the low molecular weight fraction of the Aβ oligomers was found. *Ex vivo* electrophysiological experiments revealed that the new material rescues the long-term potentiation from the toxic Aβ oligomers in mouse hippocampal slices at submicromolar concentration.

**Conclusions:**

The combination of the foldamer methodology, the fragment-based approach and the multivalent design offers a pathway to unnatural protein mimetics that are capable of specific molecular recognition, and has already resulted in an inhibitor for an extremely difficult target.

## Introduction

Unnatural self-organizing biomimetic polymers (foldamers) emerged as promising materials for protein recognition and inhibition [1]–[3]. Their tunable molecular frameworks can offer interaction surfaces to address receptors, protein-protein interactions and enzymes. Such targets are the somatostatin [4] and the transmembrane region of the integrin α_Iib_ [5] receptors, the p53-hDM2 [2], [6]–[9] and BH3-Bcl-x_L_ [10]–[13] interactions, the gp41 virus cell infusion protein assembly, [14]–[17] and the γ-secretase enzyme [18]. Foldamers may have the potential to improve on monoclonal antibodies and related protein therapeutics [19] thanks to their considerably smaller size, their bottom-up designed modular chemical structures, their resistance to hydrolysis and their tunable pharmacokinetic properties [20]–[23]. Nonetheless, it is still a major challenge to construct foldamers with a contiguous recognition surface, [24]–[28] or long sequences with broadly distributed recognition contacts [17].

In this work, foldameric recognition elements were utilized to capture the β-amyloid (Aβ) oligomer aggregates. These Aβ species correlate with the severity of Alzheimer's disease (AD) [29]–[32]. Soluble Aβ oligomers may contribute to learning and memory deficits in AD by inhibiting NMDA-receptor-dependent long-term potentiation (LTP), a cellular substrate of learning and memory.[33]–[35] Aβ oligomers [33], [36], [37] are difficult targets for various reasons: (i) their high-resolution structure is not known, (ii) they exist as transient mixtures of various species, (iii) they have a high disorder content, and (iv) the potential binding regions are exposed to the solvent. The disadvantageous properties call for an antibody approach, and a quest is currently under way for therapeutically effective neutralizing antibodies against toxic Aβ aggregates.[38]–[42] Engineered proteins have also been shown to interact tightly with various Aβ species: affibody Z_aβ3_, [43], [44] miniature protein TJ10, [45] single chain variable fragments [46], [47] and green fluorescent protein derivatives [48], [49].

Here, we discuss foldamer-based protein mimetics which were designed by following the principles of multivalent biomolecule-recognizing ligands [50]–[60]. Divalent size-selective chemical probes for Aβ oligomers [61], [62] and a tetravalent peptide-dendrimer conjugate Aβ aggregation inhibitor [63] have been reported earlier. In this work, foldamer-dendrimer conjugates were constructed with ordered recognition segments (helical foldamers) and disordered linker regions (G0-PAMAM dendrimer). This arrangement afforded wrapping of the Aβ(1–42) oligomers through the repeating binding sites displayed over the oligomeric surface. The new material rescues the long-term potentiation (LTP) from the toxic Aβ oligomers in ex vivo mouse hippocampal slices.

## Results

### Design of the foldamer segments

**Figure 1:**
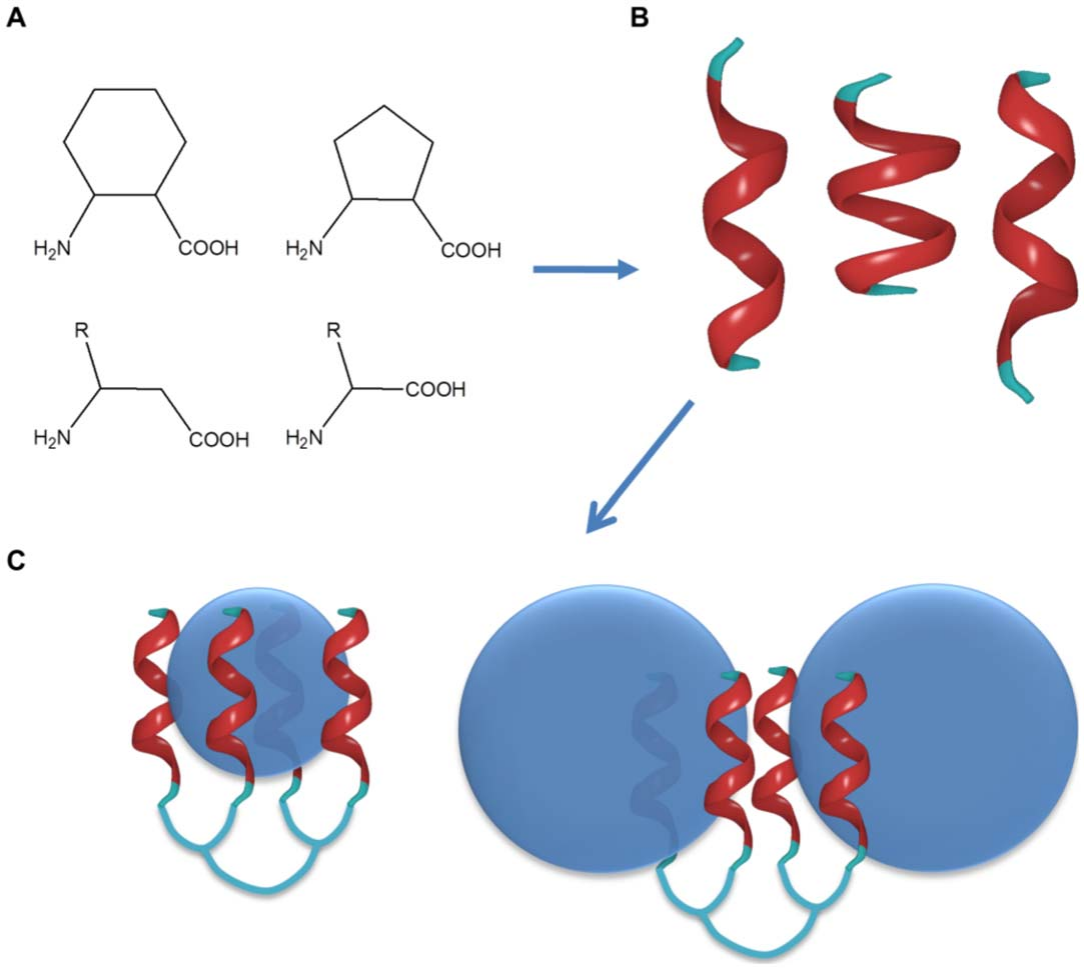
Design principles of the foldamer-dendrimer conjugates. Foldamers based on unnatural β-amino acid building blocks (R: proteinogenic side chains) fold into short helices (**A**). Foldamers exhibiting weak binding to the target can be identified by using NMR spectroscopic methods (**B**). Chemoselective ligation of the synthetic recognition segments with flexible linkers yields amplified affinity to the target (**C**). Blue spheres are schematic representation of the Aβ oligomers.

Results on peptides interacting with Aβ [64]–[70] and the structural analysis of peptide – Aβ interactions [71] suggested that the Aβ(16–22) (KLVFFAE) segment is likely to play roles in formation of the binding patch over the surface of the Aβ species. This region offers hydrophobic interactions in the core and potential salt-bridges through the flanking K16 and E22 residues. We adopted this working hypothesis for the design of the foldameric helices. Short helical β-peptide foldamers with diverse secondary structure, zwitterionic side chain pattern and hydrophobic cyclic-residues were applied as potential recognition segments in the foldamer-dendrimer conjugate ligands (Figure 1). The short foldamers can direct 3–4 side chains toward a flat protein surface, and in general, they can exhibit only weak (K_D_>10 μM) binding, which can be detected by NMR methods. The foldamer helices were synthesized by using β^3^-amino acids with proteinogenic side-chains, various diastereomers of alicyclic β-amino acids with 6- or 5- membered side chains and natural α-amino acids. The helical folds were structurally induced by using the recently published principle of backbone stereochemical patterning, so that the small-sized library contained pure β-peptidic H14, H12, H10/12 and H14/16 helices and the α, β-peptidic H9-12 helix type [72].

### Testing the foldamer – Aβ interactions by NMR

**Figure 2:**
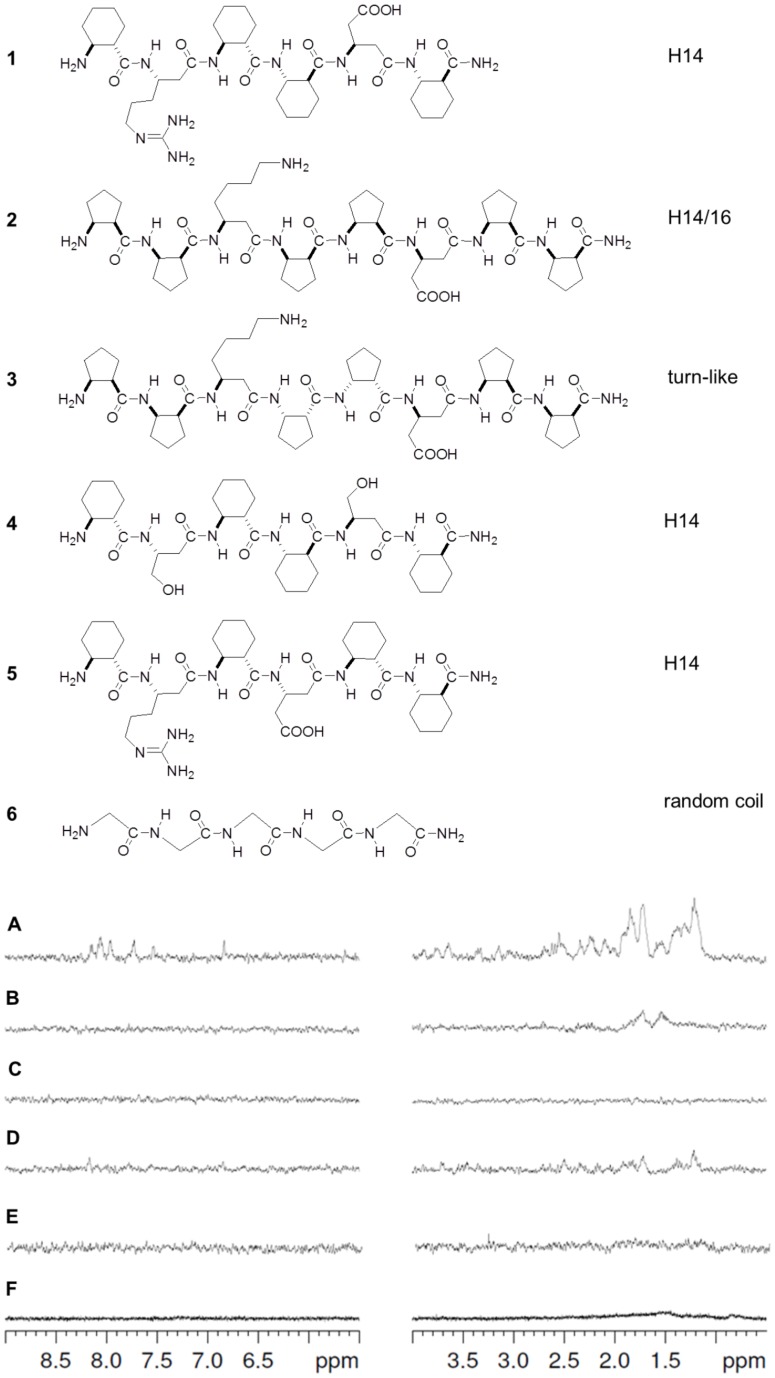
Screening for the foldameric recognition segments. Informative regions of the STD spectra (**A**–**F** for **1**–**6**, respectively) in the presence of Aβ(1–42) oligomers. The spectra were recorded in 20 mM phosphate buffer at pH 7.4, the total concentration of the Aβ(1–42) was 72 μM, and the ligands were applied at 2 mM. The secondary structure type is indicated to the right of the structures. ‘H’ stands for helix, and the numbers show the size of the H-bonded pseudocycles stabilizing the helices.

Saturation transfer difference (STD) NMR was employed to screen the weak foldamer – Aβ(1–42) oligomer interactions. For all the tests in this work, the Aβ oligomer samples were prepared by using the Ser^26^ depsipeptide aproach, [73] which furnishes the native sequence at pH 7.4 (see details in the Materials and Methods). Compound **1** exhibited well-detectable signals in the whole ^1^H-NMR spectrum (Figure 2). This was confirmed via transferred-nuclear Overhauser effect (tr-NOE) spectroscopy (Figure S1). The structure refinement indicated that this foldamer adopts an H14 helix in aqueous buffer. Thus, the β^3^-homo-Arg and β^3^-homo-Asp side chains are in *i* – *i*+3 juxtaposition. The long-range NOE interactions characteristic of the H14 helix could also be found in the tr-NOESY spectrum recorded in the presence of the target, which strongly suggested that the binding conformation of **1** is H14 helix. The structurally related **2** and **4** displayed weak saturation transfer effects only. No signal was observed for **3** supporting the importance of the helical conformation and the zwitterionic pharmacophore in the binding. To test the necessity of the proximity of the ion pair for binding, **5** was measured where the ionizable side chains point to opposite directions. For **5**, no interaction was found in STD. The virtually featureless **6** was utilized as a negative control and it did not exhibit an STD effect.

### Design and synthesis of the foldamer-dendrimer conjugates

**Figure 3:**
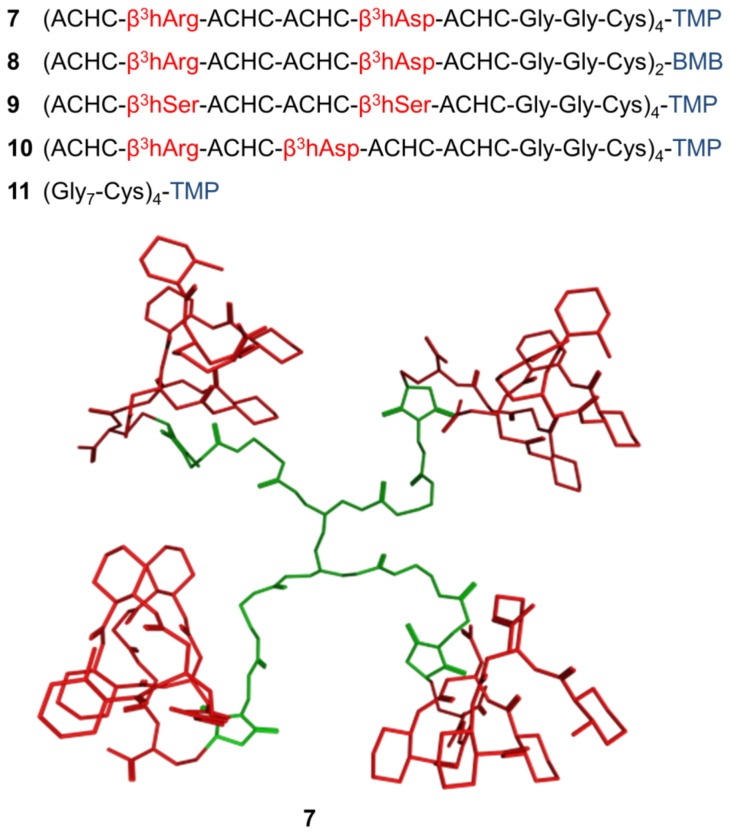
Foldamer-tetra-maleimidopropionyl-G0-PAMAM (TMP) and -bis-maleimido-butane (BMP) conjugates studied. ACHC stands for trans-2-aminocyclohexanecarboxylic acid. In the structure of **7**, the H14 helical conformation adopted by the foldamer segments in water is indicated (red), whereas the flexible linker is in an arbitrary conformation (green).

**Figure 4:**
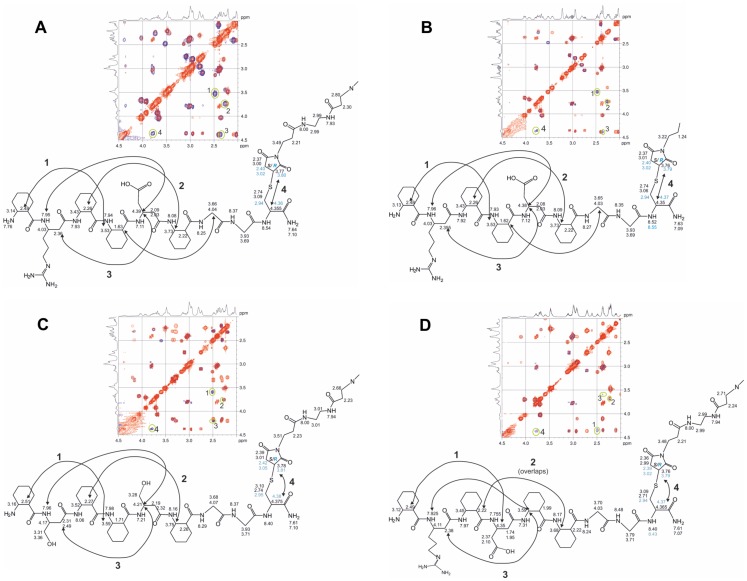
NMR assignments and long-range NOE interactions. Data are displayed for the foldamer segments and the maleimide diastereomers for **7**, **8**, **9** and **10** in panels **A**, **B**, **C** and **D**, respectively. Crosspeaks in the overlaid TOCSY (red) and ROESY (blue) spectra prove the H14 structure of the foldamers. The long-range NOEs supporting the helical conformation were observed in aqueous medium. The addition of the thiol group to the maleimido moiety generates an additional stereogenic center. The NMR resonances of the Cys-maleimide linker region are split and their integrals indicate that the addition is not stereoselective; *R* and *S* configurations can be found in equimolar ratio (*S* and *R* maleimide diastereomers are signed with black and blue, respectively). Since this undetermined configuration moiety is in the flexible part of the molecule, the effect of the chiral center does not propagate further toward either the foldamer part or the PAMAM template.

It is very likely that Aβ oligomers have a periodic structure, and binding patches for **1** are therefore displayed repetitively. Accordingly, it was expected that the tethering of **1** to a suitable multiple-armed template would lead to a tightly binding multivalent ligand. For this purpose, a generation zero poly-amido-amine (G0-PAMAM) dendrimer was employed. The C-terminus of **1** was functionalized with a Gly-Gly-Cys linker and the resulting sequence was ligated to the four-armed tetra-maleimidopropionlyl-PAMAM derivative. The foldamer-G0-PAMAM conjugate **7** is depicted in Figure 3 (for the chemical structure see Figure S2). The foldamer segment exhibited the long-range NOE interactions characteristic of the H14 helix, whereas the dendrimer moiety remained unstructured (Figure 4). The effects of multivalency on the binding properties were studied with the help of **8**, a divalent conjugate obtained by ligating **1** to a *bis*-maleimido-butane linker (Figure S2). To gain an initial insight into the structure-affinity relationship features of the tetravalent ligands, **4**, **5** and **6** were also coupled to the G0-PAMAM template, leading to compounds **9**, **10** and **11**, respectively.

### Nanomolar and stoichiometric interaction between 7 and the Aβ oligomers (ITC and DLS)

**Figure 5:**
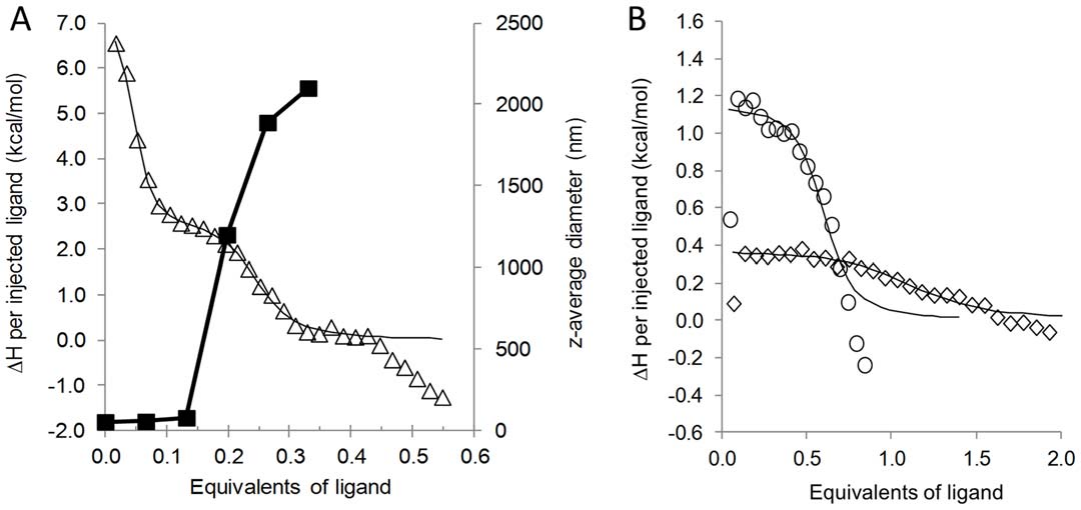
Tight binding between ligand 7 and the Aβ oligomers as determined by ITC. (**A**) ITC enthalpogram for the titration of the 72 μM Aβ oligomer with **7** up to 0.55 equivalents (triangles, left scale). Data was fitted with the two independent site model (black). The corresponding z-average diameters (squares) measured by using DLS are displayed on the right vertical scale. (**B**) ITC enthalpograms obtained for **1** (diamonds) and **8** (circles) at 72 μM Aβ oligomer concentration.

Binding of **7** to the Aβ oligomers was monitored by means of isothermal titration calorimetry (ITC). Since precipitation was observed in the ITC cell, dynamic light scattering (DLS) measurements were carried out in parallel (Figure 5A). The titrations were run at 288 K in order to improve the signal to noise ratio, and just above the precipitation limit as the ITC method is sensitive to the thermal noise caused by the stirring of large particles. After correction for the heat of ligand dilution, the sample containing 72 μM Aβ in the titration cell exhibited a two-stage enthalpogram (Figures 5A and S3). The first binding step had K_D_ = 6.9±1.4 nM, ΔH_b_ = 7.24±0.05 kcal mol^−1^ and N = 0.041±0.00089, whereas the lower affinity interaction was characterized by K_D_' = 281.1±38.7 nM, ΔH_b_' = 2.58±0.02 kcal mol^−1^ and N' = 0.18±0.002. These values strongly supported tight binding between the Aβ oligomer species and **7**. DLS data (Figure 5A) revealed that the first binding event did not affect the average particle size, while the precipitation started just before the equivalence point of the second stage.

### Effects of multivalency on the binding affinities as detected by ITC and ELISA

The ITC titration with **8** resulted in a single-stage enthalpogram (Figure 5B, K_D_ = 721.4±120.1 nM, ΔH_b_ = 1.1±0.12 kcal mol^−1^ and N = 0.53±0.003) and the parameters correlated well with those measured for **7** in the second stage. The rather low endothermic ΔH_b_ for **1** made fitting difficult, but the stoichiometry clearly increased to ∼1∶1 (N = 0.97±0.05) and K_D_ increased above 2 μM. Neither **8** nor **1** led to concentration-dependent precipitation.

**Figure 6:**
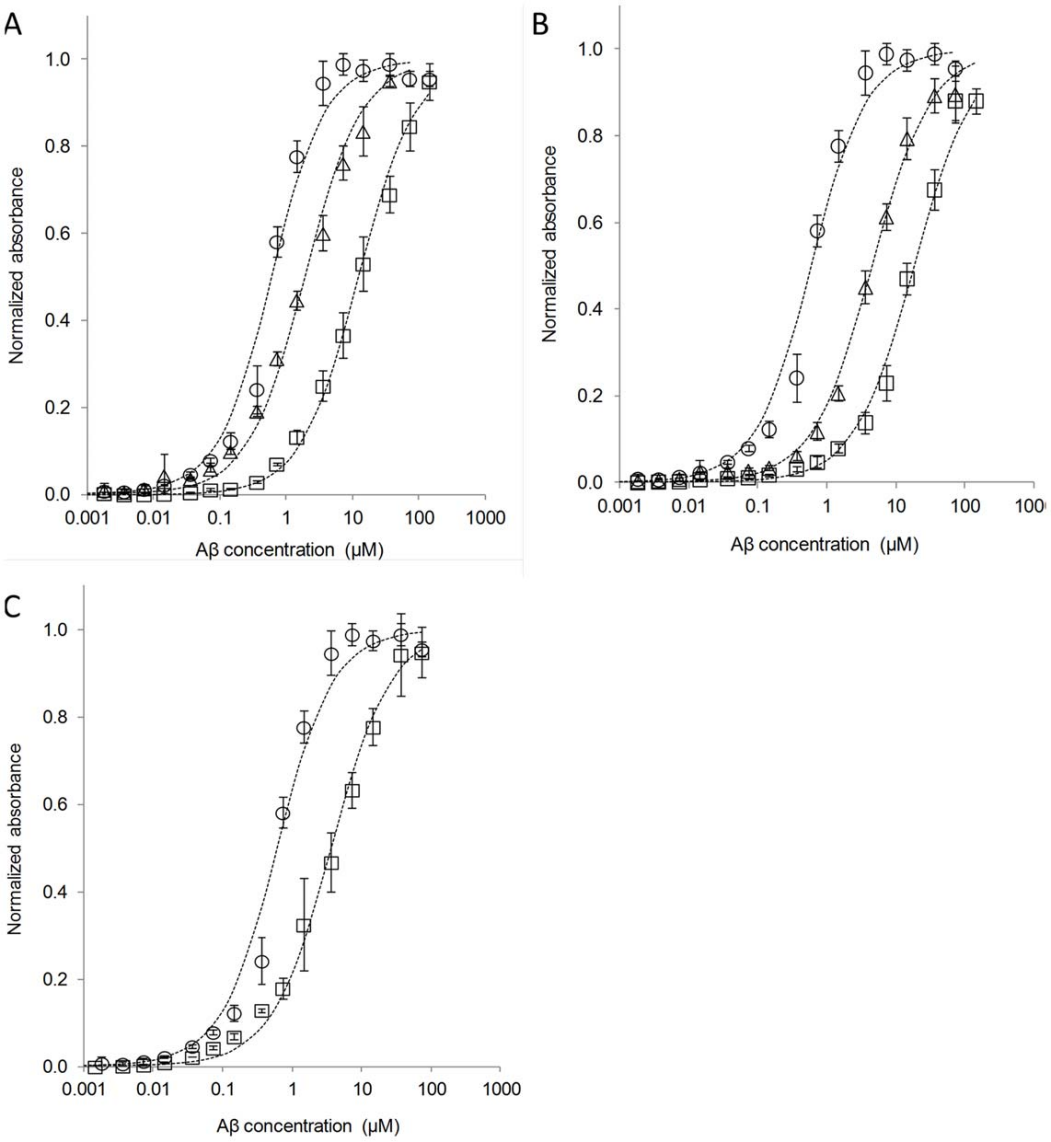
ELISA results. (**A**) Effects of multivalency on the affinities. Normalized absorbances recorded in capture ELISA experiments, where the biotinyl-**7** (circles), -**8** (triangles) or -**1** (squares) are attached to the streptavidin functionalized plate and the increasing surface concentration of Aβ is observed through the BAM10 antibody. Fitted curves are given (dashed), IC50 values are 126 nM, 933 nM and 12 µM for biotinyl-**7**, -**8** and -**1**, respectively. (**B**) ELISA curves measured for biotinyl-**7** (circles), -**9** (triangles) and -**10** (squares) with oligomeric Aβ and the IC50 values are 126 nM, 4.4 µM and 18.5 µM, respectively. (**C**) ELISA curves recorded for oligomeric Aβ (circles) and fibrillar Aβ (squares) with biotinyl-**7**, the IC50 values are 126 nM and 886.2 nM, respectively.

Biotin-labeled **1** was prepared by elongating the foldamer segment with a biotinyl-aminohexanoyl-Gly-Gly moiety and these derivatives were coupled to G0-PAMAM which furnished biotin-labeled **7**. Biotinyl-**8** was also prepared. All the labeled ligands were attached to the streptavidin functionalized microplates with a coverage of 5 pmoles per well. The ELISA datasets (Figure 6A) revealed that biotinyl-**7** successfully captured Aβ oligomer species at nanomolar affinity, even on the solid support. Because of the potential sterical shielding of the recognition segments over the surface, the ITC affinities cannot be directly compared to the ELISA results. The IC50 values for biotinyl**-8** and **-1** exhibited the same increasing trend as observed for the apparent K_D_-s in the ITC titrations.

These results demonstrated the effects of multivalency and strongly suggested that low nanomolar binding and affinity precipitation were possible only with **7** in this set. Divalent **8** still exhibited submicromolar affinity, but it was not able to initiate ligand-induced precipitation. The stoichiometric (1∶1) binding observed for **1** indicated that one binding patch is formed per an Aβ chain, and these interaction sites are all available on the surface. ITC titration with monomeric Aβ sample did not exhibit submicromolar binding suggesting that a certain aggregation level is necessary for the tight and stoichiometric binding of **7**.

### Initial structure-affinity relationship tests on the foldamer-dendrimer conjugates

ITC measurements with **9**, **10** and **11** did not reveal any tight binding to the Aβ oligomer species (Figure S4). This phenomenon was tested also in a capture ELISA. Biotin-labeled **4** and **5** were prepared as described and ligation to G0-PAMAM yielded biotin-labeled **9** and **10**, respectively. ELISA measurements confirmed the ITC findings (Figure 6B). The high-affinity interaction tolerated neither the removal of the ionic side-chains (**9**) nor the changing of the position of the β^3^-hAsp residue (**10**), which supported the selective nature of the interaction. Interestingly, the ELISA curve recorded with fibrillar Aβ and biotinyl-**7** also indicated decreased affinity suggesting the size-selective nature of the nM interaction (Figure 6C).

### Selective low nM interaction of 7 with the LMW fraction of Aβ oligomers

**Figure 7:**
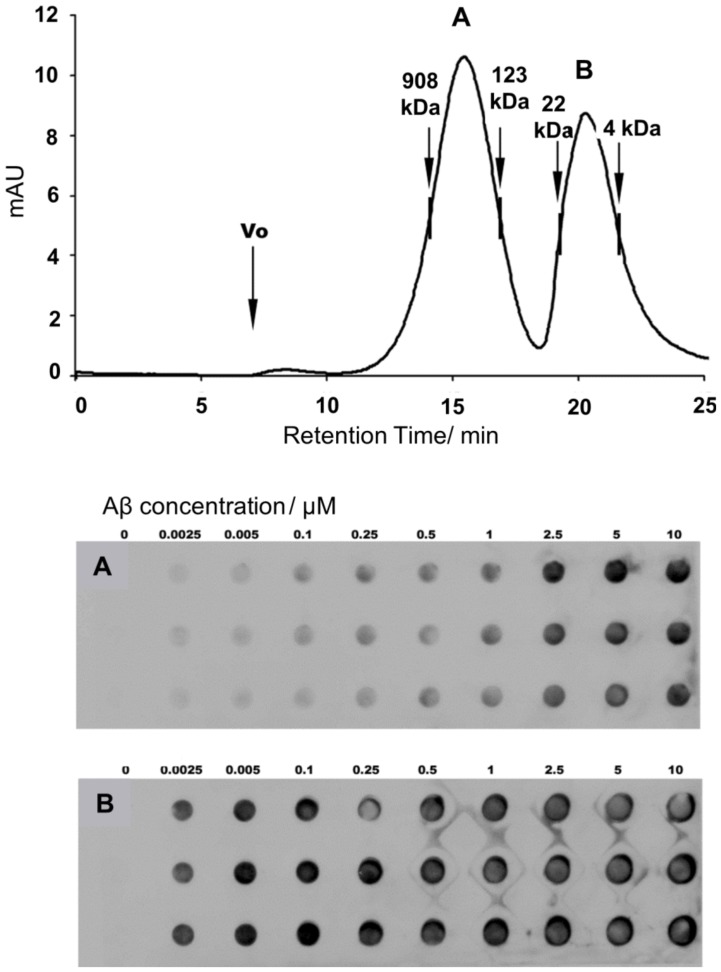
Selective binding to the LMW Aβ oligomers in the nM range. Size exclusion chromatographic separation of the HMW and LMW fractions of the Aβ oligomeric sample (top panel). Concentration-dependent dot blot experiments performed with the HMW (**A**) and the LMW fractions (**B**). The ligand loadings were 10 µg aliquots of **7**, and the sequence specific antibody BAM10 (1∶500) was utilized. Lanes within the panels are parallel experiments.

ITC measurement for **7** indicated fractional stoichiometry for the low nM binding, which pointed to that the high affinity interaction involves only a fraction of the oligomeric Aβ sample. To localize the strongly binding fraction in the mixture, size exclusion chromatographic (SEC) separation was carried out and the resulting Aβ oligomer fractions were tested with **7** in concentration-dependent dot blot experiments. Two dominant peaks were found in the SEC chromatogram corresponding to the high molecular weight (HMW) and low molecular weight (LMW) components (Figure 7). The LMW fraction displayed saturation of binding in the low nanomolar region, whereas HMW species were stained with just below micromolar affinity. In control experiments, specific and/or preferential binding of Aβ by the membrane itself were ruled out. These findings were in line with the ITC and ELISA results. It could be concluded that **7** preferentially bound to the LMW fraction at low nM concentration and also interacted with the HMW fraction at sub-µM level.

### Effects of the binding on the particle size and on the secondary structure of the oligomeric Aβ

**Figure 8:**
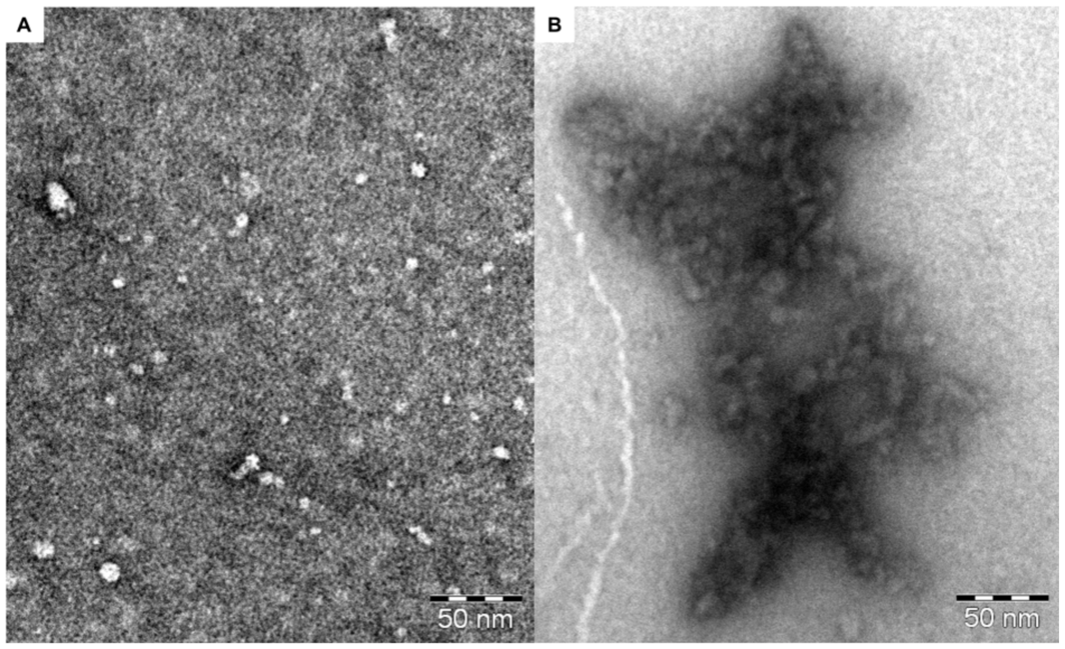
Affinity precipitation as detected by TEM. **(A)** TEM image of the 72 µM Aβ oligomer. (**B**) TEM image of the 72 µM Aβ oligomer upon addition of 36 µM **7**.

The concentration-dependent increase of the particle size described above raised the question if this phenomenon was linked to an accelerated fibrillization or to any form of conformational remodeling of the Aβ oligomers. The TEM (transmission electron microscopy) images (Figure 8), revealed that **7** caused association of the spherical oligomers a few nm in size into bundles of aggregates in the μm range. The shape of the aggregates was unequivocally different from that of the mature fibrils.

**Figure 9:**
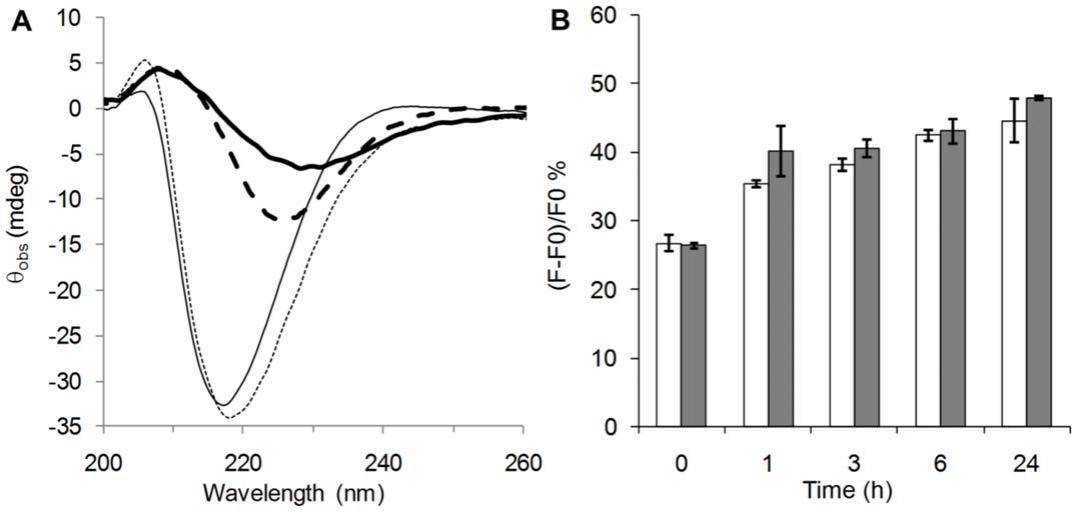
No change was observed in the Aβ secondary structure. (**A**) Observed ellipticities in ECD for 36 μM Aβ oligomer (thick black), 9 μM **7** (thin black), and 36 μM Aβ +9 μM **7** (thick dashed). The hypothetical sum of the ECD curves of the pure samples (thin dashed). (**B**) ThT binding experiment carried out on 36 µM oligomeric Aβ (white bars) and on 36 µM oligomeric Aβ +**7** 1∶1 mixtures. The relative fluorescence values were monitored up to 24 h.

The electronic circular dichroism (ECD) results indicated that the pure Aβ oligomer solution contained a mixture of β-sheet and random coil conformations (Figure 9A). The data on **7** were in agreement with its largely helical structure, also found via NMR. Mixing 0.25 equivalents of **7** with 36 μM Aβ solution did not lead to an immediate increase in the β-sheet content. As the conformation of the ligand concerns, the intensity of the negative lobe decreased significantly at around 220 nm, but a significant change was not observed below 210 nm. This suggested that **7** retained its helical conformation content since minor geometry changes can alter the band intensities for short β-peptidic foldamers significantly, while disordering would have caused a blue shift. This was in accord with the NMR results obtained for the recognition segment **1**. The secondary structure was probed also with ThT binding experiments (Figure 9B). Pure Aβ oligomers bound ThT at a certain level. This was in accord with the staining with antibody OC; both are capable of recognizing the fibrillar (or protofibrillar) oligomers. Compound **7** had no significant effect on the ThT binding. The biophysical characterization by using TEM, ECD and ThT binding confirmed that conformational remodeling did not occur upon binding: disaggregation of the oligomers into random coil structure was not observed and the particle size increase was due to the non-covalent cross-linking with **7**. The latter process is similar to affinity precipitation.

**Figure 10:**
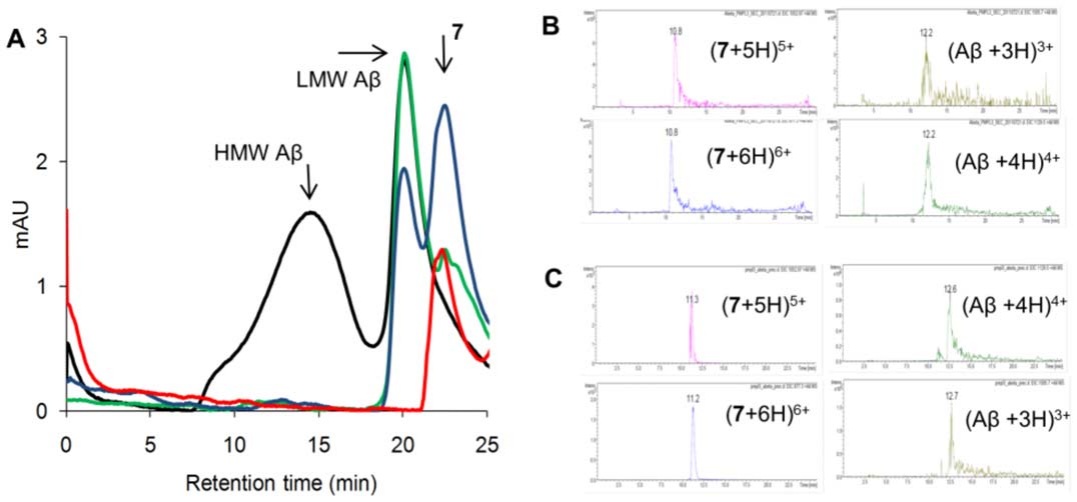
Lower tendency of the LMW Aβ oligomer fraction to affinity precipitation. (**A**) SEC analysis of pure Aβ oligomers (black) and mixtures of 50 µM Aβ oligomers with **7** in 0.25 (green) and 1.0 (blue) equivalents. The samples were injected on the SEC column after filtration. The control SEC chromatogram of **7** was recorded (red) and it exhibited anomalously longer retention time due to its compact geometrical arrangement. (**B**) LC-MS results on the LMW fraction taken at 19 min. Both Aβ and **7** were identified in the chromatogram in a comparable amount. (**C**) LC-MS results on the HMW precipitate, which confirmed the heterocomplex nature of the product.

The higher affinity of **7** toward the LMW oligomer fraction and the lack of size-selectivity for the dimeric **8** suggested that the LMW fraction, possibly binding more than two tentacles of **7**, exhibits a decreased tendency to the affinity precipitation. This hypothesis was tested via SEC analysis (Figure 10A). Mixtures of 50 µM Aβ oligomers with **7** in 0.25 and 1.0 equivalents were injected on the SEC column after filtration. At 0.25 equivalents, the HMW fraction fully precipitated and disappeared from the SEC chromatogram, whereas the LMW fraction remained intact. At 1.0 equivalents, the HMW fraction again disappeared and a partial decrease was observed for the LMW fraction. We concluded that the affinity precipitation occurs for the HMW component, but the LMW fraction is also affected at higher ligand concentrations. The control SEC chromatogram of **7** was recorded and it exhibited anomalously longer retention time due to its compact geometry. Thus it was possible to test if the LMW fraction co-elutes with **7**. LC-MS was run on the LMW fraction taken at 19 min and both Aβ and **7** were identified in the chromatogram in a comparable amount (Figure 10B). The HMW precipitate was also tested for the presence of **7** with LC-MS, and the result confirmed the heterocomplex nature of the product (Figure 10C).

**Figure 11:**
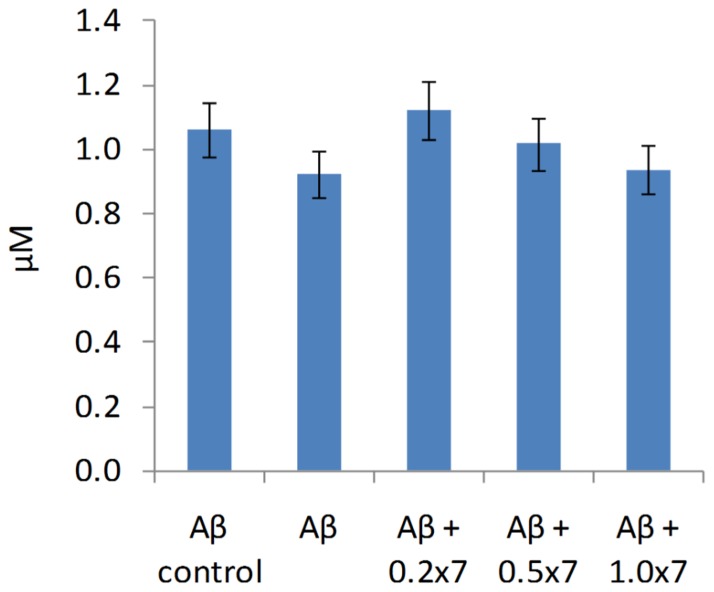
Aβ oligomers did not precipitate out of the solution at 1 µM. Aβ concentrations determined by ELISA in supernatants obtained from samples containing 1 µM Aβ oligomer mixed with 0, 0.2, 0.5 and 1.0 equivalents of **7** and centrifuged at 15000×g (room temperature, 3 h).

The extent of precipitation was also tested at an Aβ oligomer concentration of 1 µM. The Aβ oligomer sample was mixed with 0, 0.2, 0.5 and 1.0 equivalents of **7**, centrifuged at 15000× g for 3 h and the supernatant was tested for residual Aβ concentration by using a standard ELISA (Figure 11). This measurement displayed no significant loss of soluble material at 1 µM, which facilitates biological experiments at this concentration without titrating Aβ out of the solvent upon adding **7**.

### Foldamer-dendrimer conjugate 7 rescues LTP *ex vivo*

We investigated whether **7** could protect against the synaptic plasticity damage caused by Aβ(1–42) oligomers by using a hippocampus slice LTP model. LTP, a correlate of learning and memory has been repeatedly shown to be impaired by Aβ(1–42) oligomers. As negative control, substance **11** was applied. The experimenters were blind to the compounds tested.

**Figure 12:**
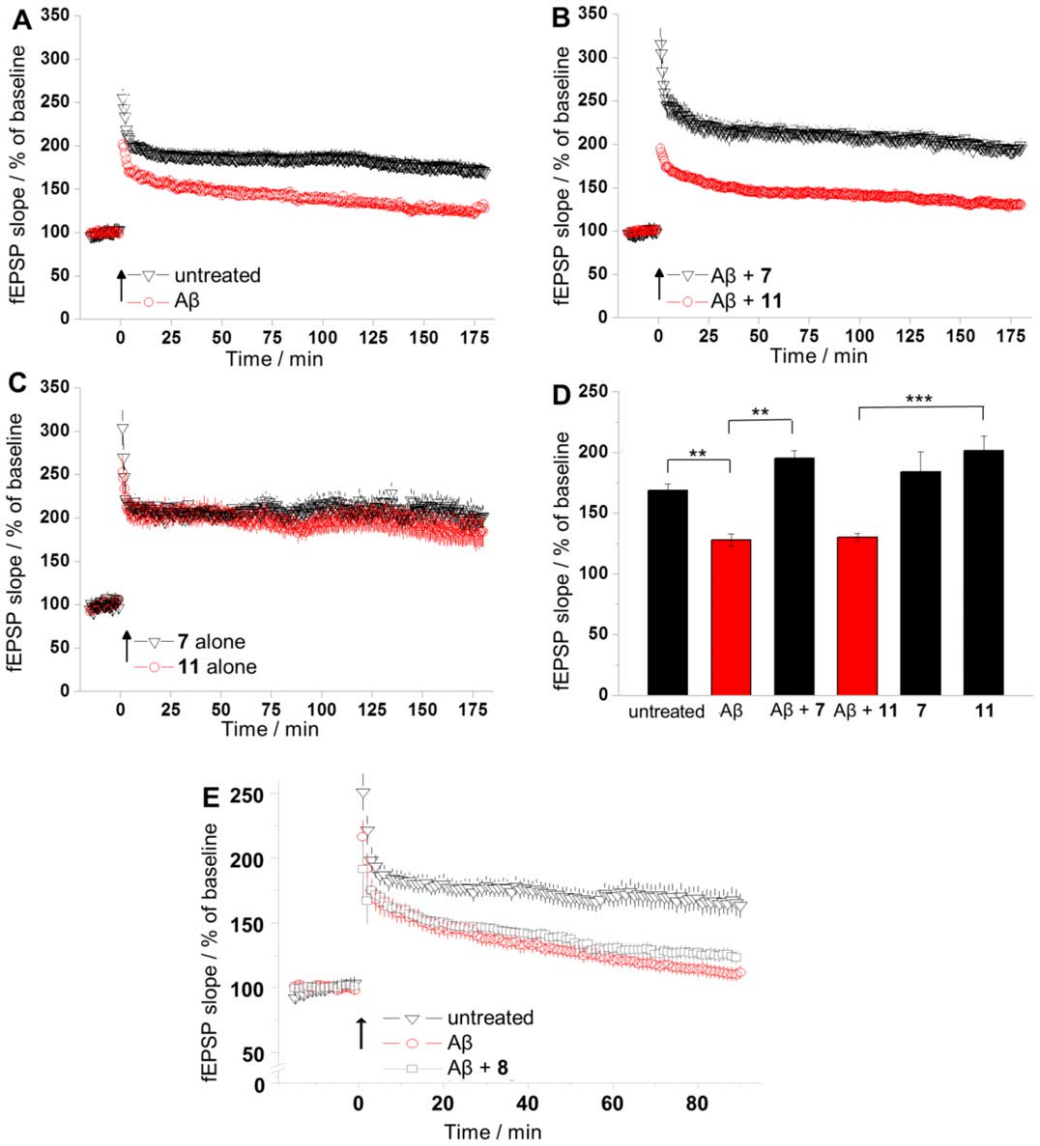
Ligand 7 protects against Aβ-induced LTP impairment. (**A**) The oligomeric Aβ(1–42) sample applied at 720 nM hinders synaptic potentiation. (**B**) Compound **7** applied at 950 nM prevents the LTP impairment caused by Aβ(1–42) oligomers. The control substance **11** has no effect against Aβ(1–42) oligomers. (**C**) Neither **7** nor **11** alone exerted any effect on LTP in the absence of Aβ oligomers. (**D**) The summarized results observed 180 min after LTP (**P<0.01, ***P<0.001 versus control). Statistical analysis was carried out by using two-tailed Student's t-test, n = 6 or 7 slices per group. Data are presented as means ±SEM. (**E**) Divalent **8** applied at 950 nM did not exhibit statistically significant effect against Aβ(1–42) oligomers. Arrows indicate LTP induction, EPSP stands for excitatory postsynaptic potential.

Untreated slices exhibited a robust potentiation (175±5%, n = 7, Figure 12A), and LTP was reduced by the Aβ(1–42) oligomer at 720 nM (128±5%, n = 7, p = 0.003). The application of Aβ(1–42) oligomers and **7** (950 nM) together resulted in normal LTP (195±5%, n = 6, Figure 12B), but the control with Gly_7_ tentacles (**11**) did not lead to rescue from the toxic effect (130±3%, n = 6, p≤0.001 vs. **11** alone). Importantly, neither **7** nor **11** alone exerted any effect on LTP (184±16%, n = 6 and 200±14%, n = 6, respectively) which indicates that the protective effect is Aβ-dependent (Figure 12C). These observations indicate the significant protective effect of **7** (Figure 12D). To test the effects of valency, LTP experiments were repeated with **8** under the same conditions. No rescue from the toxic Aβ oligomers was observed for **8** (Figure 12E).

## Discussion

Foldameric helices were designed based on the assumption that an organized hydrophobic surface flanked by zwitterionic side chains may lead to ligands binding to the Aβ oligomeric species. The NMR screening carried out on foldamers with various secondary structures resulted in that a H14 helix built with 2-aminocyclohexanecarboxylic acid (ACHC) residues and ionizable β^3^-amino acids (**1**) is indeed capable of weak binding to the target. The initial structure affinity relationship study suggested that the pharmacophore is well defined; changing arrangement/nature of the hydrophobic side-chains, removal of the zwitterionic feature and the distance between the charged side-chains abolish binding. In order to amplify affinity, a tetravalent foldamer-dendrimer conjugate (**7**) was designed and synthesized. Various methods revealed that **7** captures Aβ oligomers stoichiometrically with submicromolar affinity, and a selective low nanomolar interaction with the LMW oligomer fraction was observed. Due to the multivalent nature of the ligand, affinity precipitation occurred at higher target concentrations. For the divalent **8**, neither the low nM binding affinity toward the LMW Aβ oligomers nor the affinity precipitation occurred. Moreover, the reduced ligand-induced precipitation tendency for the LMW fraction strongly suggests that the low nM binding requires more than two or possibly all the recognition segments of **7**. The stoichiometric binding of the ligands and the effects of multivalency may carry important information on the solution structure of the Aβ oligomers. This may open up new directions in the structural analysis of the Aβ oligomers.

Designed Aβ binding proteins [43]–[49] have been shown to capture monomeric Aβ as demonstrated by X-ray and SPR studies (carried out on immobilized monomeric Aβ). This type of interaction arrested the aggregation process and the formation of the toxic species could be avoided. The multivalent structures containing the KLVFF segment [61]–[63] target the LMW oligomers through assumed incorporation into the β-sheet structure. The selective interaction with LMW oligomers have been demonstrated for dimeric structures [62]. The aggregation process could also be inhibited this way, but protecting effects against the toxic Aβ oligomers have not been described.

In *ex vivo* measurements, **7** provided rescue from the LTP inhibiting effect of the Aβ oligomers at submicromolar concentrations in an Aβ-dependent manner, whereas **8** was not active. The protective effect was immediate on the time scale of the LTP measurements; it did not require preincubation of the Aβ oligomer samples with the ligand. As concerns the mechanism of action, our results do not support any conformational remodeling in the Aβ oligomer mixture at the ligand concentration applied (e.g., shifting the equilibrium toward monomeric Aβ). Many protective agent cited above have been shown to act as aggregation inhibitor, but the mechanism is different in this case. The tight and stoichiometric binding of **7** to the target may efficiently block the toxic surface of the Aβ oligomers which leads to the LTP rescue.

Methods to protect synaptic plasticity from the neurotoxic species have been described in the literature. These interventions improve specific synaptic processes of LTP damaged by Aβ via CaMKII activation and subsequent AMPA receptor phosphorylation [74] or reduction of mitochondrial superoxide formation [75]. The corresponding compounds act on general routes without intercepting and neutralizing Aβ, whereas anti-Aβ antibodies have also been shown to rescue hippocampal LTP *in vivo* [76]. We followed the latter strategy with a synthetic protein mimetic.

In general, the described approach offers a pathway to unnatural molecules that are capable of specific molecular recognition, and has already resulted in an inhibitor for an extremely difficult target. Although this new class of bioactive materials has potential advantages over certain protein therapeutics, further studies will be necessary to test its therapeutic and/or diagnostic utility with a special focus on the ability of crossing the blood-brain-barrier.

## Materials and Methods

### Ethics Statement

All animal experiments were conducted according to the ethics statement and approval of the National Ethics Committee for Animal Experimentation and of the Ethics Committee of University of Szeged (approval no.: XXVII./03405/2008). The animals were kept and the experiments were conducted in conformity with Council Directive 86/609/EEC and with the Hungarian Act of Animal Care and Experimentation (1998, XXVIII).

### Preparation of the toxic Aβ(1–42) oligomer samples

Preparation of the synaptotoxic Aβ(1–42) oligomers: In this work, a depsipeptide derivative of Aβ(1–42) was utilized, [73] which was converted to the native sequence by applying physiological pH. At pH 7.4, oligomers are spontaneously formed without the application of any detergent or residual organic solvent in the final sample. The Ser^26^ depsipeptide *iso*-Aβ(1–42) was synthesized and purified as reported earlier [73]. The standardized protocol for the preparation of the toxic oligomer samples was as follows. The lyophilized *iso*-Aβ(1–42) was treated with HFIP for 24 h, after which the organic solvent was thoroughly removed *in vacuo*. The resulting material was dissolved in MilliQ water to a concentration of 1 mM, sonicated for 10 min and diluted into PBS buffer (pH 7.4) to nominal concentration of 100 μM. The sample was incubated at 37°C for 24 h. The peptide content of the final sample was determined by amino acid analysis to be 72%. This oligomeric stock solution was either applied directly or diluted to the required concentration with PBS prior to use. The aggregation grade was monitored with various techniques; the toxicity was proved in *ex vivo* LTP experiments.

### Characterization of the Aβ(1–42) oligomer samples

#### Conformation-specific antibodies

Staining with the conformation-specific antibody A11 [77], [78] gives negative reaction, whereas the positive reaction with antibody OC [79] indicated the protofibrillar nature of the oligomers.

#### SEC

The size exclusion chromatogram revealed two peaks (Figures 7 and 10) in the size ranges of 8–22 kDa and 123–908 kDa, which correspond to the low molecular weight (LMW) and the high molecular weight (HMW) oligomer populations.

#### SDS-PAGE and Western Blot

We also examined the size distribution of the oligomers by means of conventional denaturing SDS-PAGE (Figure S5), applying antibodies BAM10 and OC for staining. The fresh sample contained mainly monomers and LMW oligomers, whereas after 24 h, HMW oligomers were present besides the LMW fraction and the monomer population was undetectable. Staining with antibody OC confirmed the fibrillar (protofibrillar) nature of the oligomers.

#### ThT binding

The relative fluorescence values were monitored up to 24 h, and the results indicate that Aβ oligomers bind ThT at a certain level (Figure 9B). This is in accord with the staining with antibody OC, both capable of recognizing the fibrillar (or protofibrillar) oligomers.

#### ECD

In order to gain information on the conformation of the oligomers formed, ECD curve was analysed, and a mixture of β-sheet and disordered structures were found (Figure 9A). These indicate the protofibrillar fibrillar nature of these oligomers.

#### TEM

The TEM measurements (Figure 8) revealed the size of the globular oligomers in their dehydrated and stained state. The oligomers were observed to be spherical with an average diameter of 7.4±3.3 nm. Detailed analysis of the sizes demonstrated the presence of two size classes: the smaller oligomers possessed an average size of 4.9±1.0 nm (d_min_ = 2.8 nm, d_max_ = 6.9 nm), and the larger ones a size of 10.4±2.4 nm. Again, the presence of two oligomer populations could be assumed. Monomers could not be observed in the TEM images.

#### DLS

Dynamic light scattering (DLS, Figure S6) measurements furnished a size distribution curve of the oligomers, where the frequencies of the different sizes were normalized to the intrinsic volume of the scattering particles. The primary frequency data provided by the DLS measurements were somewhat distorted, as the contribution of a scattering particle to the total measured intensity of the scattered light is directly proportional to the sixth power of its size. Normalization of the frequencies to the intrinsic volume therefore provides a distribution with less distortion. This method can be applied only when the scattering particles are spherical; the TEM images proved the spherical nature of the Aβ(1–42) oligomers. The DLS data reflected a bimodal size distribution curve, demonstrating that the oligomer population was heterogeneous. The maximal values (10.1 nm and 37.8 nm) were relatively large, but it should be considered that the measured values were hydrodynamic diameters, for the fully hydrated state of the peptide assemblies, together with the solvent shell of the particle. The ratio of the frequencies of the two size classes was ∼ 4∶1, indicating, that the small oligomers were overrepresented as compared with the large assemblies. Noteworthy is that the presence of large scattering particles could suppress the contribution of the small ones to the total intensity, which caused an artifact: monomers can not be observed if large oligomers are present in the sample.

#### NMR

^1^H-NMR spectra (Figure S7) were recorded for the starting Ser^26^ depsipeptide *iso*-Aβ(1–42) at pH 3 and after buffering the medium to pH 7.4. *iso*-Aβ(1–42) at acidic pH exhibits narrow peaks, indicating that it exists mostly in monomeric form. On switching to the native sequence through setting of the physiological pH, the total peak intensity dropped to 30%, as oligomer species with MW > ∼100 kDa (d>∼3–4 nm) were outside the detection limit of the solution phase ^1^H-NMR for T2 relaxation reasons. The narrow peaks can be assigned to the residual monomeric and possibly LMW oligomeric (N<4) fractions.

### Foldamer synthesis

Foldamers were synthesized on solid support with standard Fmoc-chemistry. Foldamers and biotinyl-**1** were synthesized on a Tentagel R RAM resin (0.17 mmol g^−1^) on a 0.1 mmol scale with 1-[bis(dimethylamino)methyliumyl]-1*H*-1,2,3-triazolo [4,5-*b*]pyridine-3-oxide (HATU) as coupling reagent. The octapeptide (Gly)_7_ Cys-amide was synthesized by conventional Fmoc-based SPPS on Rink-amide resin, applying DCC/HOBt activation. The amino acid incorporation was monitored by means of the Kaiser test and by the cleavage of aliquots from the resin. The peptide sequences were cleaved from the resin with cocktail of TFA/H_2_O/DTT/TIS (90∶5∶2.5∶2.5) at room temperature for 3 h. The TFA was removed *in vacuo*, and the peptide was precipitated in dried diethyl ether. The resulting free peptide precipitate was filtered off, were dissolved in 10% aqueous acetic acid, and lyophilized. The crude peptide was purified by RP-HPLC on a Phenomenex Luna 10 μ column (10 mm x 250 mm). The solvent system consisted of 0.1% TFA in water (A), and 0.1% TFA in 80% acetonitrile (B); the default gradient was 0% – 40% B during 15 min, and then 40% – 70% during 60 min at a flow rate of 4 mL min^−1^, with detection at 206 nm. The gradient was customized where necessary.

### Synthesis of the tetra-maleimidopropionyl-PAMAM conjugate

300 µl 25 wt% methanol solution of polyamidoamine dendrimer generation 0, ethylenediamine core (0.4 mmol, Sigma Aldrich) was lyophilized for 1 h to remove methanol. The resulting oily substance was dissolved in 1 ml DMF and added dropwise to a mixture of 1.2 mmol maleimidopropionic acid, 248 mg DCC and 163 mg HOAt dissolved in DMF. The mixture was stirred for 4 h at ambient temperature, diluted with water and lyophilized to dryness. Prior to purification, the white powder was dissolved in a mixture of ACN (80%)/TFA (0.1%)/H_2_O, sonicated for 10 min, diluted with 0.1% TFA/H_2_O solution, filtered through a glass filter and injected onto a Phenomenex Luna C18 (250×10 mm, 100 Å, 5 µm) semipreparative HPLC column, applying ACN (5–50%)/TFA (0.1%)/H_2_O gradient elution at 3.0 ml min^−1^ flow rate. ESI-MS spectrum: [MH^+^]: 1121.69; [MH_2_^2+^]: 561.43 (calculated MW: 1121.3) For characterization of the materials see Figures S7, S8, S9, S10, S11.

### Synthesis of 7-11 and biotinyl-7-11

4 µmol (20.0 mg) *N,N,N,N*-tetra-maleimidopropionyl-PAMAM(G0) was dissolved in 8 ml 50 mM NaH_2_PO_4_/Na_2_HPO_4_ buffer (pH = 7.1). 19.2 µmol peptide (**1-**Gly-Gly-Cys, biotinyl-**1-**Gly-Gly-Cys or (Gly)_7_Cys-amide) was dissolved in 1 ml of the same solution, and added dropwise to the dendrimer under constant stirring. The reaction was stirred for 4 h at ambient temperature, then deep-frozen and left to stand overnight at −20°C. The following day, the mixture was injected directly onto a Phenomenex Jupiter C4 (250 x 10 mm, 300 Å, 10 µm) semipreparative HPLC column and purified by ACN (0–70%)/TFA (0.1%)/H_2_O gradient elution at 3.0 ml min^−1^ flow rate. The material content of the lyophilized **7**, determined by thermogravimetry, was 95%.

Compound **8** was synthesized the same way, but bis-maleimido-butane linker was utilized for the ligation.

### Transmission electron microscopy

Oligomer solution was placed on formvar-carbon-coated 400-mesh copper grids (Electron Microscopy Sciences, Washington, PA) and stained negatively with uranyl acetate. The aggregates were characterized by TEM on a Philips CM 10 transmission electron microscope (FEI Company, Hillsboro, Oregon, USA) operating at 100 kV. Images were taken with a Megaview II Soft Imaging System, routinely at magnifications of ×46000 and ×64000, and analyzed with an AnalySis® 3.2 software package (Soft Imaging System GmbH, Münster, Germany).

### Dynamic light scattering (DLS)

For DLS measurements, 500 µL Aβ(1–42) solution (c = 72 µM) was prepared in PBS, and placed in a low–volume sizing cell. Size distribution was measured at 25°C on a Malvern Zetasizer Nano ZS Instrument (Malvern Instruments Ltd. Worcestershire, UK) equipped with a He-Ne laser (633 nm) by means of Non-Invasive Back Scatter (NIBS®) technology, which involves detection of the scattered light at an angle of 173°. A titration routine was formulated consisting of 12 independent measurements with a 2-min delay after each. The calculated amount of **7** solution was added to the Aβ after every second measurement. During the titration, the molar quantity of **7** varied between 0–35 µM. For a single measurement, the correlation function and distribution of the apparent hydrodynamic diameter (d_h_) over the scattered intensity of the particles were determined on the basis of 14 scans. The translational diffusion coefficients were obtained from the measured autocorrelation functions by using a fitting algorithm incorporated in the software package Dispersion Technology Software 5.1 (Malvern Instruments Ltd. Worcestershire, UK).

### NMR spectroscopy

NMR spectra were recorded on a 600 MHz Bruker Avance spectrometer equipped with a 2.5 mm triple-resonance capillary probe. The protein and the ligands were dissolved in 20 mM, pH 7.4 phosphate buffer (90% H_2_O, 10% D_2_O) containing 0.02% NaN_3_. Spectra were acquired with the WATERGATE solvent suppression pulse scheme. For the STD and tr-NOE measurements, the Aβ(1–42) and the ligand concentrations were 100 μM and 2.0 mM, respectively. As a reference, STD and tr-NOE experiments were also performed without the target, containing the ligand alone.

STD spectra were acquired by using a series of 40 equally spaced 50-ms Gaussian-shaped pulses for selective saturation of the protein, with a total saturation time of 2 s. The frequency of the on-resonance saturation was set at −1 ppm and the off-resonance saturation frequency was set at 40.0 ppm. A total of 2 k scans were collected for each pseudo 2D experiment. The 2D tr-NOESY measurements were performed with 128 increments and 256 scans, with a NOE mixing time of 200 ms.

Signal assignments were performed by using the 2D TOCSY and ROESY spectra of the 2 mM samples recorded at 298 K in aqueous buffer and d3-MeOH. Because of the better signal resolution at lower temperatures, the spectra of **7** in buffer were acquired at 280 K. ROESY measurements were carried out with a mixing time of 400 ms and with 32 and 64 scans. TOCSY measurements were acquired with homonuclear Hartman–Hahn transfer with the MLEV17 sequence, with a mixing time of 80 ms; the number of scans was 32. For all the 2D spectra, 2048 time domain points were applied, while the number of increments was 128 and 512 for **1** and **7**, respectively. The processing was carried out by using a cosine-bell window function, with single zero filling and automatic baseline correction.

### Molecular mechanics and NMR structure refinement

Molecular mechanical simulations were carried out in the Molecular Operating Environment (MOE) of the Chemical Computing Group. For the energy calculations, the MMFF94x force field was used, without a cut-off for van der Waals and Coulomb interactions. The implicit water model of GB/VI (Generalized Born) was applied. The conformational sampling was carried out by using the hybrid MC/MD simulation (as implemented in MOE) at 300 K with a random MC sampling step after every 10 MD steps. The MC-MD was run with a step size of 2 fs for 20 ns, and the conformations were saved after every 1000 MD steps, which resulted in 10000 structures. For the NMR-restrained simulation, the upper distance limits were calculated by using the isolated spin-pair approximation and classified by following the standard method (strong: 2.5 Å, medium: 3.5 Å, and weak: 5.0 Å). The lower limit was set at 1.8 Å. Restraints were applied as a flat-bottomed quadratic penalty term with a force constant of 5 kcal Å^−2^. The final conformations were minimized to a gradient of 0.05 kcal mol^−1^ Å^−1^) and the minimization was applied in a cascade manner, using the steepest-descent, conjugate gradient and truncated Newton algorithm.

### Isothermal titration calorimetry

Isothermal titrations were performed with a Microcal VP-ITC microcalorimeter. The binding experiments were performed in PBS pH 7.4. The buffer solution was degassed. The concentrations of the ligands and Aβ were corrected for the material content. In individual titrations, 10 μL of ligand was injected from the computer-controlled 300-μL microsyringe at intervals of 300 s into the Aβ oligomer solution dissolved in the same buffer as the ligand. The microsyringe stirring was set to 295 rpm. All measurements were made at 288 K. The Aβ concentrations in the cell were either 72 μM or 36 μM, and the concentrations of **7** were 175 μM and 222 μM respectively. The total ligand concentration was set in the syringe so that the titration stopped when the precipitation became excessive. Control experiments were performed by injecting the ligand into a cell containing buffer with no target, and the heats of dilution were subtracted from those measured in the presence of Aβ. Titrations were also performed with buffer in the syringe and Aβ oligomer sample in the cell to check for the heat response of Aβ dilution itself. The dilution heat of the Aβ oligomer samples was constant and negligible. Since the target is inherently inhomogeneous, and an *a priori* model of the binding events is not available,. the experimental data were fitted to the two independent site binding model by using a nonlinear least-squares procedure, with ΔH_b_, ΔH_b_', K_a_, K_a_' (association constants), N and N' (number of binding sites for monomer), as adjustable parameters. The exothermic peaks appearing after the fully precipitated phase was reached, were omitted from the regression. The results are summarized in Table S1.

### Electronic circular dichroism

CD spectra were measured on a Jasco J815 dichrograph in a 1.00 cm cell using PBS (pH = 7.4) as solvent. Three spectra were accumulated for each sample. The baseline spectrum recorded with only the solvent and it was subtracted from the raw data.

### ELISA

An indirect ELISA experiment was conducted on 96-well streptavidin-coated clear plates (Pierce, Cat.No. 15500, Rockford, IL, US). Biotinyl-compounds bound to streptavidin by incubating 1 µg substance per well for 2 h at ambient temperature. Aβ(1–42) was dissolved in PBS to 100 µM and incubated for 24 h at 37°C. Serial dilutions of the incubated peptide were prepared, and supplemented with Tween (0.05% v/v of the final volume) and BSA (1% w/v of the final volume) prior to use. Compounds were incubated with Aβ oligomers for 1 h at ambient temperature. Bound Aβ was detected by monoclonal anti-Aβ AB clone Bam-10 (Sigma-Aldrich) applied in 1∶10000 dilution for 1 h followed by an incubation with anti-mouse IgG-HRP (DakoCytomation, Glostrup Denmark). Finally, 100 µL tetramethylbenzidine (TMB) solution (Cell Signaling Tecnology Inc., Danvers, USA) was introduced into the wells, and the change in the absorbance at 370 nm was monitored constantly, without the addition of stop solution, on a 96-well plate reader (NOVOstar OPTIMA, BMG Labtech, Offenburg, Germany) equipped with a xenon lamp, fiberglass optics and a shaking microplate carrier. Abs_370_ values were read near complete saturation of the signal intensity, which was observed after 40 min. For the quantitative analysis of the results, nonlinear regressions were carried out and the IC50 values were optimized with fixed number of blocked ligands per Aβ peptide as determined by ITC [80]. For **7**, the iteration converged to IC50  = 126 nM (N = 0.25), and marked deviations from the experimental curve were found indicating a substoichiometric higher affinity binding. For **8** and **1**, the IC50 values were 933 nM (N = 0.5) and 12 µM (N = 1.0). Concerning the steric shielding exerted by the solid support, the apparent binding parameters estimated from ELISA are in good accordance. Measurements with fibrillar Aβ and **7**, revealed an affinity similar to the value obtained for the oligomeric Aβ – **8** interaction, which may suggest that tetravalent **7** can attach to the fibrillar Aβ only as a divalent ligand.

### Size Exclusion Chromatography

*iso*-Aβ(1–42) was dissolved in sodium hydrocarbonate buffered saline (pH 7.4) to 50 µM and incubated for 24 h at 37°C. The oligomeric Aβ was loaded onto an Äkta Purifier FPLC system (GE Healthcare, UK) equipped with a Superose 6 10 300 column. Sample was eluted at a flow rate of 0.5 ml/min. FPLC chromatogram was taken at 280 nm and 1 ml fractions were collected during the separation. Fractions corresponding to the chromatographic peaks (No.10–12 for peak **a** and 16–18 for peak **b**) were pooled and their peptide content was equalized with running buffer according to their concentration determined by UV measurement at 220 nm.

### SDS-PAGE and Western Blot

10-μL aliquots of the Aβ oligomer samples were loaded onto a 15% SDS-polyacrylamide gel without boiling prior to loading, while 5 μL Kaleidoscope Precision Plus Protein ^TM^ (Bio-Rad Laboratories, Ca, USA) standard was applied as MW marker. After running, the gel was transferred to a nitrocellulose membrane using an electroblotting apparatus (Bio-Rad Laboratories, CA, USA). The membrane was blocked in TBS, 0.1% Tween-20 (TBST), 5% non-fat dry milk and incubated overnight with primary antibody (1∶1000 OC (Millipore) or 1∶2500 Bam-10 (Sigma) at 4°C. The following day the membranes were incubated with an anti-mouse-HRP secondary antibody (1∶10000; DakoCytomation, Glostrup, Denmark) and Pierce ECL Western Blotting Substrate (PIERCE, Rockford, IL). Blots were exposed to Kodak film (Sigma).

### Dot Blot experiment: binding study

10 µg aliquots of **7** dissolved in TBS were spotted onto a 0.1 µm nitrocellulose membrane with a Dot Blot apparatus (Bio-Rad Laboratories, Ca, USA). The spots were washed in the instrument with 2×200 µl TBS. After serial dilutions of both oligomer fractions **a** and **b**, 100 µl aliquots were loaded in the wells and incubated with gentle shaking for 1 h at RT. The membrane was washed twice with TBS, blocked with a blocking solution (5% BSA, 0.1% Tween-20 in TBS, 2×30 min) and incubated with the sequence-specific Bam-10 (Sigma) antibody in 1∶500 dilution overnight at 4°C. After washing of the membrane twice with blocking solution, the secondary antibody (HRP-conjugated anti-mouse AB; Dako Cytomation, Glostrup, Denmark) was incubated with the membrane in 1∶10000 dilution (2 h at 4°C) followed by washing twice with blocking solution and twice with 0.1% Tween-20 in TBS. 8. The membrane was incubated in ECL Western Blotting Substrate (Pierce) for 1 min and exposed to Kodak film (Sigma).

### ThT binding measurements

ThT (Sigma-Aldrich) was dissolved in 50 mM NaH_2_PO_4_/Na_2_HPO_4_ buffer (pH = 7.0) to a final concentration of 25 μM. This working solution was kept at 4 °C protected from light and used during the measurement. Two identical 50 μM Aβ oligomer samples were prepared and one of them was mixed with **7** to a final concentration of 50 μM, and the ThT signal intensity was monitored up to further 24 h. At each time point, samples were carefully vortexed to get a homogeneous sample and to resuspend the precipitated material, and 50 μL aliquots were mixed with 500 μL of working solution. ThT-peptide mixtures were vortexed again and 150 μL aliquots were placed on a 96 well plate. ThT fluorescence was measured on a plate reader at λ_ex_ =  m and λ_em_ = 480 nm. Data mean values and S.D. were calculated from three parallel measurements.

### Sample preparation and ELISA for concentration determination of Aβ in the precipitation study

1 μM solution of the Aβ oligomer sample was prepared. This solution was divided to 4×15 ml fractions and **7** was added to three samples in 1, 0.5 or 0.2 μM final concentration, respectively. All the four samples were divided into three fractions (2×5 ml). One fraction from each sample was centrifuged at 15000×*g* at RT for 3 hours in a Roth MIKRO 200 Microcentrifuge (Carl-Roth GmbH Karlsruhe, Germany) centrifuge. One fraction from each sample was let undisturbed at RT in the meantime. After the centrifugation 3×1 ml supernatant was removed from each fraction and subjected with the unseparated ones to concentration determination by ELISA.

Different amounts of Aβ (0.0001, 0.0003, 0.001, 0.003, 0.01, 0.03, 0.1, 0.3 µg pro well) were coated in the wells (6 parallels for each concentration) of a 96 well plate. The supernatant of the centrifuged samples was diluted two times and 10–10 µl was added to the empty wells. The plate was incubated for 20 h at 4°C, using a coating solution (15 mM Na_2_CO_3_, 35 mM NaHCO_3_, 3 mM NaN_3_). The wells were then blocked using a blocking solution (10 mM NaHCO_3_, 0.45% NaCl, 0.1% Tween-20, 1% BSA) at room temperature. After blocking, the plate was washed three times with a washing buffer (10 mM NaHCO_3_, 0.45% NaCl, 0.1% Tween- 20). A monoclonal Aβ antibody (BAM10, SIGMA) was added to the wells in blocking solution for 1 h at room temperature. After washing twice, the wells were incubated with HRP-conjugated anti-mouse secondary antibody (Dako Cytomation, Denmark, Glostrup) in blocking solution. The plate was washed two times, and TMB reagent (Cell Signaling Technology) was added to the wells. Without using a stopping reagent, the absorbance at 370 nm was constantly monitored using a FLUOstar OPTIMA Multidetection Microplate Reader (BMG LABTECH, Offenburg, Germany). Data values were read at the saturation point of the signal curves. The supernatants were analysed according to the concentration calibration.

### Hippocampal slice electrophysiology

Via standard procedures, 350-µm-thick transverse hippocampal slices were prepared from the brain of 7-months-old mice (CD1, Animal Breeding Facility, University of Szeged) with a McIlwain tissue chopper (Campden Instruments, Loughborough, UK). Slices were incubated in standard artificial cerebrospinal fluid (ACSF) at ambient temperature for 60 min, during constant gassing with 95% O_2_–5% CO_2_. The ACSF contained (mM): NaCl, 130; KCl, 3.5; CaCl_2_, 2; MgCl_2_, 2; NaH_2_PO_4_, 0.96; NaHCO_3_, 24; and D-glucose, 10 (pH 7.4). Individual slices were transferred to a 3D-MEA chip with 60 tip-shaped and 60-μm-high electrodes spaced at 100 μm (Ayanda Biosystems, S.A., Lausanne, Switzerland). The slice was continuously perfused with oxygenated ACSF (1.5 ml min^−1^ at 34 °C) containing 720 nM Aβ(1–42) oligomers and/or 950 nM ligand throughout the recording session. Data were recorded with a standard, commercially available MEA setup (Multi Channel Systems MCS GmbH, Reutlingen, Germany). The Schaffer-collateral was stimulated by injecting a biphasic current waveform (−100/+100 μs) through one selected electrode at 0.033 Hz. Care was taken to choose the stimulating electrode in the same region from one slice to the other. The peak-to-peak amplitudes of fEPSPs at the stratum pyramidale and stratum radiatum of CA1 were analyzed. After a 30-min incubation period, the threshold and the maximum of stimulation intensity for evoke responses was determined. To evoke responses, 30% of the maximal stimulation intensity was used. LTP was induced by applying a theta-burst stimulation (TBS) pattern at the maximal stimulation intensity. The TBS comprised four trains administered at 20-s intervals with 10 bursts given at 5 Hz per train and 4 pulses at 100 Hz per burst. Statistical analysis was carried out by using two-tailed Student's t-test. For representative raw data see Figure S12.

## Supporting Information

Figure S1 **TOCSY spectrum (A) and ROESY spectrum (B) of 1.** **NMR-derived conformation of 1: H14 helix (C).** tr-NOESY recorded on the mixture of **1** and the Aβ(1–42) oligomers and the NOE crosspeaks supporting the H14 helical binding conformation (**D**).
(TIF)

Figure S2 **Constitutions of 7 and 8.**
(TIF)

Figure S3 **Representative raw ITC data obtained with the 72** **µM Aβ(1–42) oligomer in the titration cell and 175** **μM 7 in the syringe.** The curve was corrected for the heat of dilution of the ligand, and polynomial baseline correction was applied.
(TIF)

Figure S4 **ITC enthalpograms for the titration of the 72** **μM Aβ oligomer with 9 (A), 10 (B) and 11 (C).** Fitting of the titration curves revealed weak (K_D_>2 µM) and substoichiometric interactions for both **9** and **10**. This indicated that these changes in the recognition segments lead to the loss of tight and specific binding. For **11**, the curve fitting did not converge, because the exothermic heat response (negative ΔH values) with negative slope cannot be associated with a binding equilibrium.
(TIF)

Figure S5 **SDS-PAGE and Western Blot characterization of the Aβ oligomer sample.** The results on the left (BAM10) and the right (OC) panels were obtained on identical samples. The incubation time was measured from dissolving iso-Aβ in the pH 7.4 buffer. The monomeric fraction is not stained by OC, whereas BAM10 has a limited efficiency in staining the LMW oligomers. The OC staining revealed that the monomeric population can be minimized with the incubation time and after 24 h a mixture of LMW and HMW oligomers was obtained.
(TIF)

Figure S6 (**A**) DLS measurement: hydrodynamic diameter distribution of the Aβ oligomers in PBS, c = 72 μM, after incubation for 24 h. Frequencies are normalized to the intrinsic volume of the scattering particles. (**B**) TEM image of the oligomers on formvar-carbon coated grids, stained with uranyl acetate, visualized at 92000× magnification.
(TIF)

Figure S7 **^1^H-NMR spectra recorded for the Ser^26^ depsipeptide** ***iso*** **-Aβ(1–42) at pH 3 (A) and after buffering of the medium to pH** **7.4 for the same sample (B).** The intensities are corrected for the small dilution.
(TIF)

Figure S8 **Purity and integrity of 7, 11 and biotinyl-7.** Analytical HPLC chromatograms of the purified **7**, **11** and biotinyl–**7** are given in panels (**A**), (**B**) and (**C**), respectively. Conditions: solution A: 0.1% TFA in water; B: 80% ACN, 0.1% TFA in water Applied gradients: **7**: 0–20% B in 20 min; **7**: 40–64% B in 12 min, biotinyl-**7**: 25–75% B in 25 min Column Phenomenex Luna 5 C18 column 1.2 ml/min flow rate at ambient temperature.
(TIF)

Figure S9 **Purity and integrity of 7, 11 and biotnyl-7. ESI-MS spectra of the purified 7, 11 and biotinyl-7 are given in panels (A), (B) and (C), respectively.**
(TIF)

Figure S10 **^1^H-NMR WATERGATE spectra recorded in H_2_O**∶**D_2_O 90**∶**10 (buffer pH** **7.4) for 7 (A) and 11 (B).** The signal broadening in the amide region is due to the chemical exchange with solvent protons. Signal broadening in methanol was not observed. Inset displays spectrum of **7** in d3-MeOH.
(TIF)

Figure S11 **^1^H-NMR WATERGATE spectra for 7–11 (A–E, respectively) recorded in H_2_O**∶**D_2_O 90**∶**10 (phosphate buffer pH** **7.4).**
(TIF)

Figure S12 **Superimposed raw data before (black) and 180** **min after (red) LTP induction (A), untreated; (B), Aβ(1–42) oligomer; (C), Aβ(1–42) oligomer +7; (D), Aβ(1–42) oligomer +11).**
(TIF)

Table S1 **Affinities to Aβ oligomers determined for 1, 7, 8, 9 and 10 with ITC and ELISA.**
(DOC)
