# Supplementary figures and images for: A Foldamer-Dendrimer Conjugate Neutralizes Synaptotoxic β-Amyloid Oligomers
Source: PLoS One. 2012 Jul 30;7(7):e39485. doi: 10.1371/journal.pone.0039485 (PMC3408453; doi:10.1371/journal.pone.0039485)

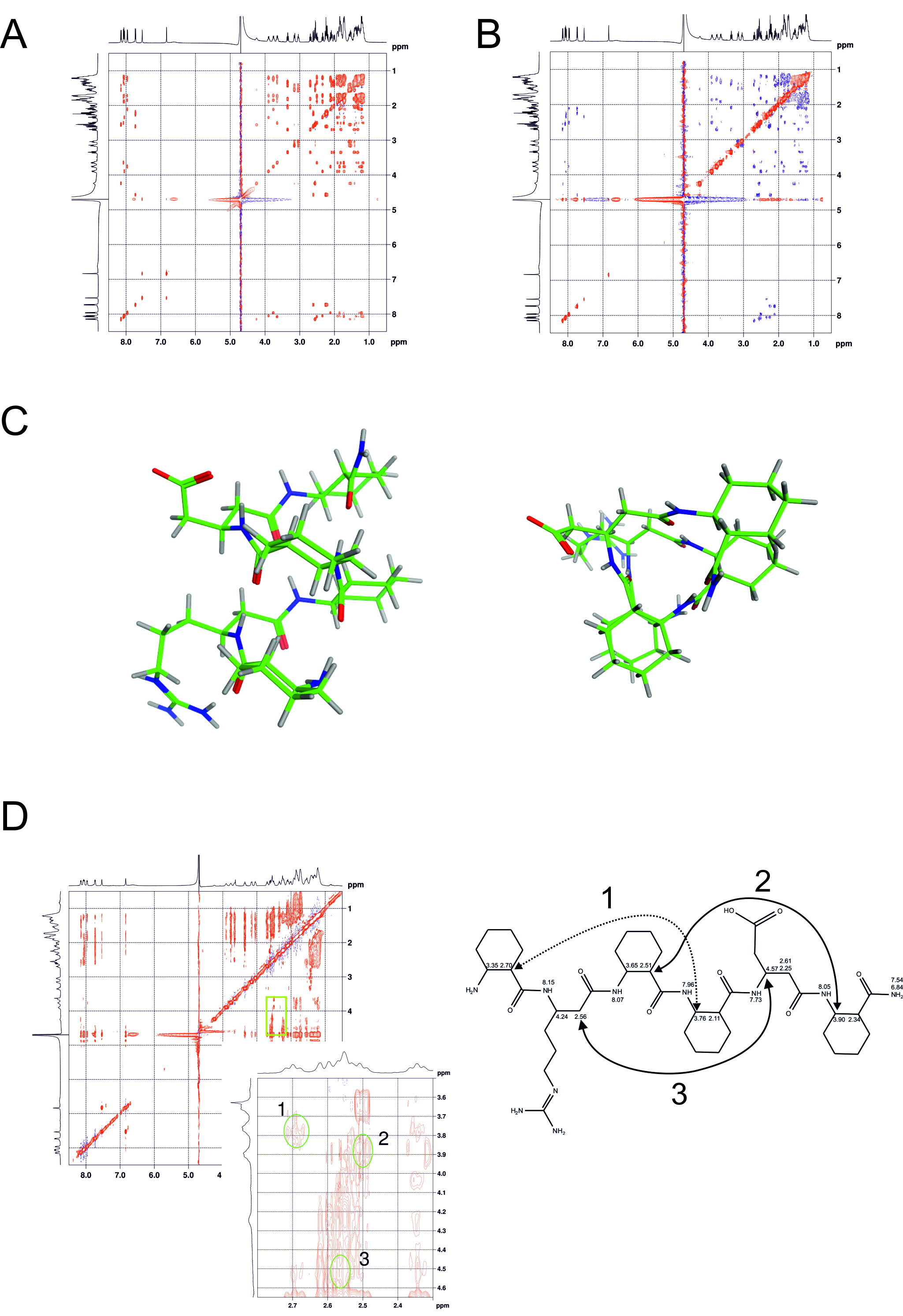

Supplement: Figure S1 — TOCSY spectrum (A) and ROESY spectrum (B) of 1. NMR-derived conformation of 1: H14 helix (C). tr-NOESY recorded on the mixture of 1 and the Aβ(1–42) oligomers and the NOE crosspeaks supporting the H14 helical binding conformation (D). (TIF) [file pone.0039485.s001.tif]

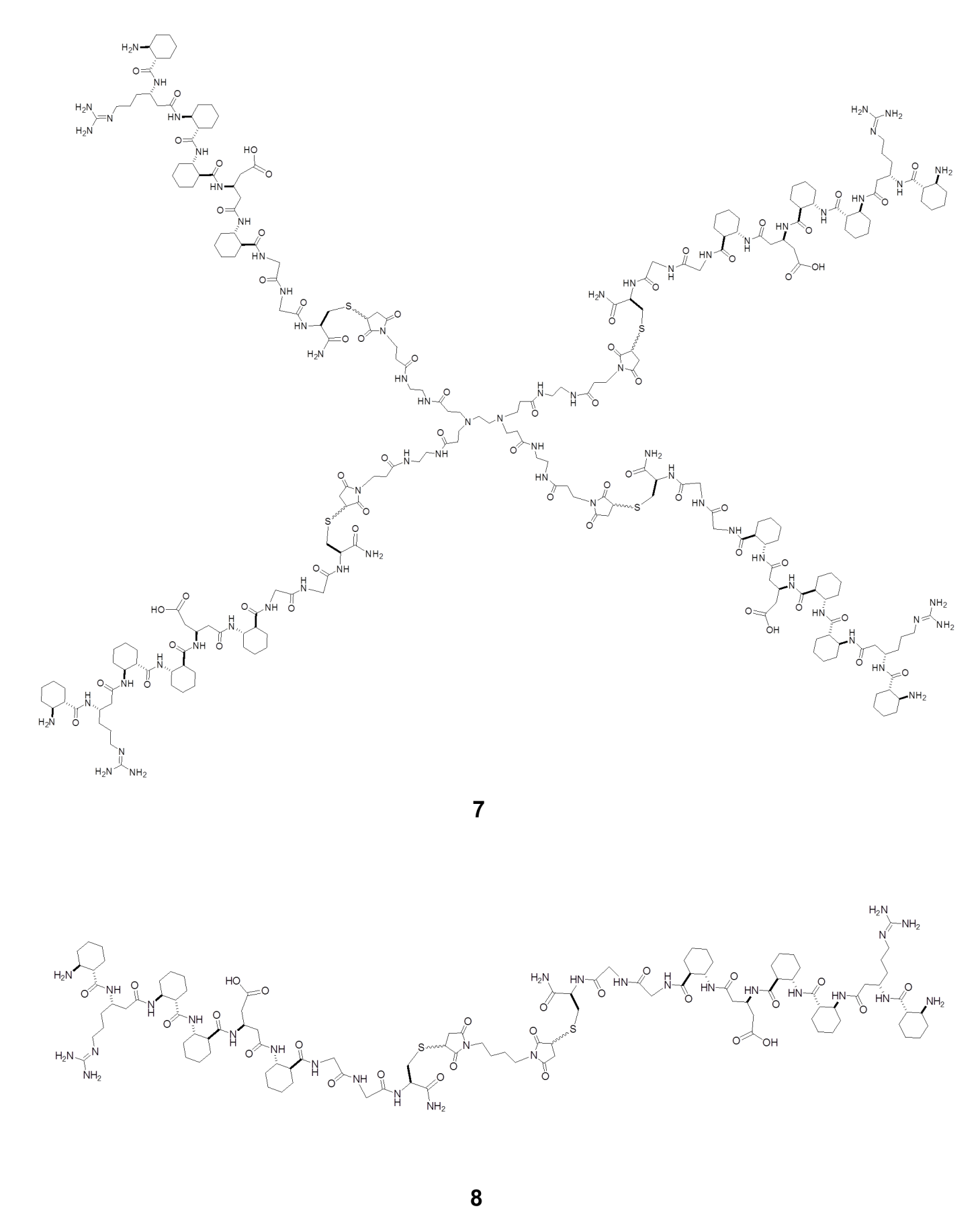

Supplement: Figure S2 — Constitutions of 7 and 8. (TIF) [file pone.0039485.s002.tif]

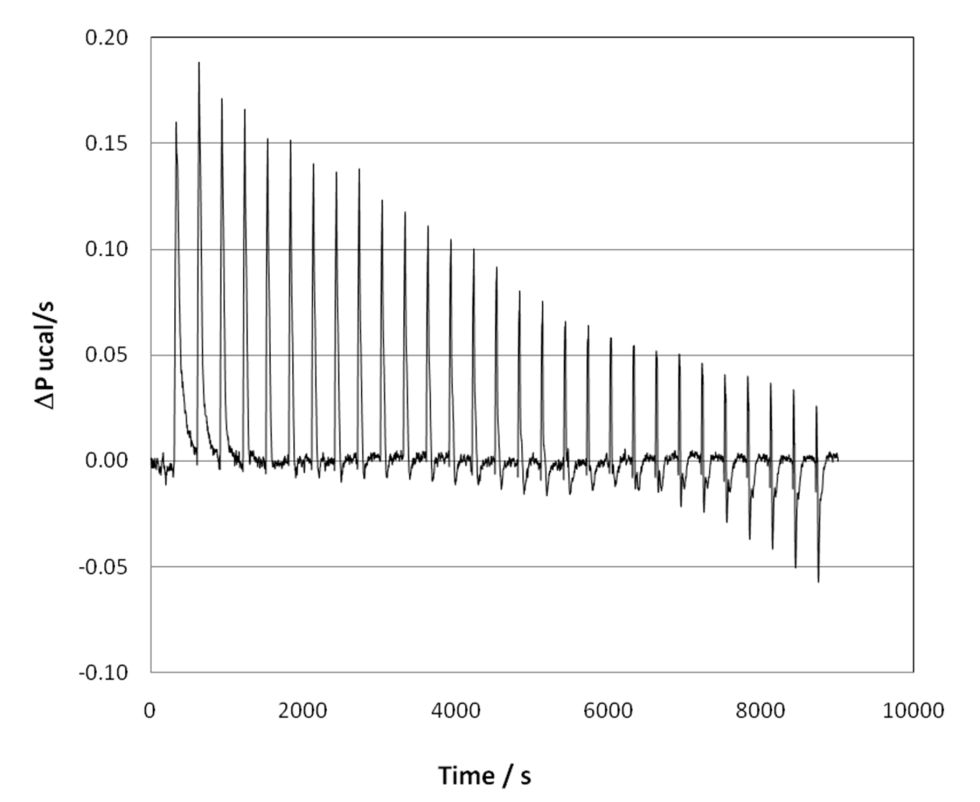

Supplement: Figure S3 — Representative raw ITC data obtained with the 72 µM Aβ(1–42) oligomer in the titration cell and 175 μM 7 in the syringe. The curve was corrected for the heat of dilution of the ligand, and polynomial baseline correction was applied. (TIF) [file pone.0039485.s003.tif]

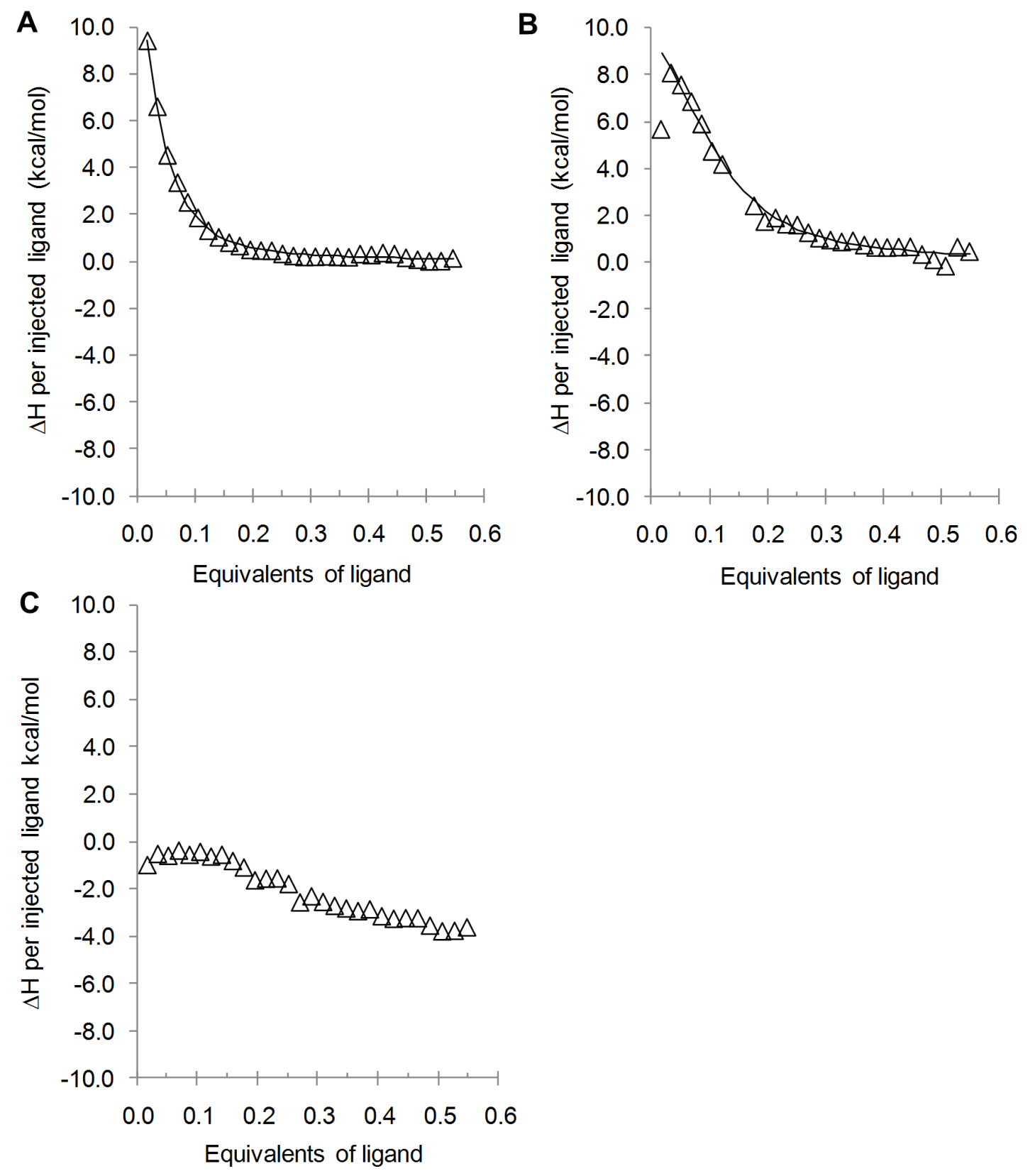

Supplement: Figure S4 — ITC enthalpograms for the titration of the 72 μM Aβ oligomer with 9 (A), 10 (B) and 11 (C). Fitting of the titration curves revealed weak (KD>2 µM) and substoichiometric interactions for both 9 and 10. This indicated that these changes in the recognition segments lead to the loss of tight and specific binding. For 11, the curve fitting did not converge, because the exothermic heat response (negative ΔH values) with negative slope cannot be associated with a binding equilibrium. (TIF) [file pone.0039485.s004.tif]

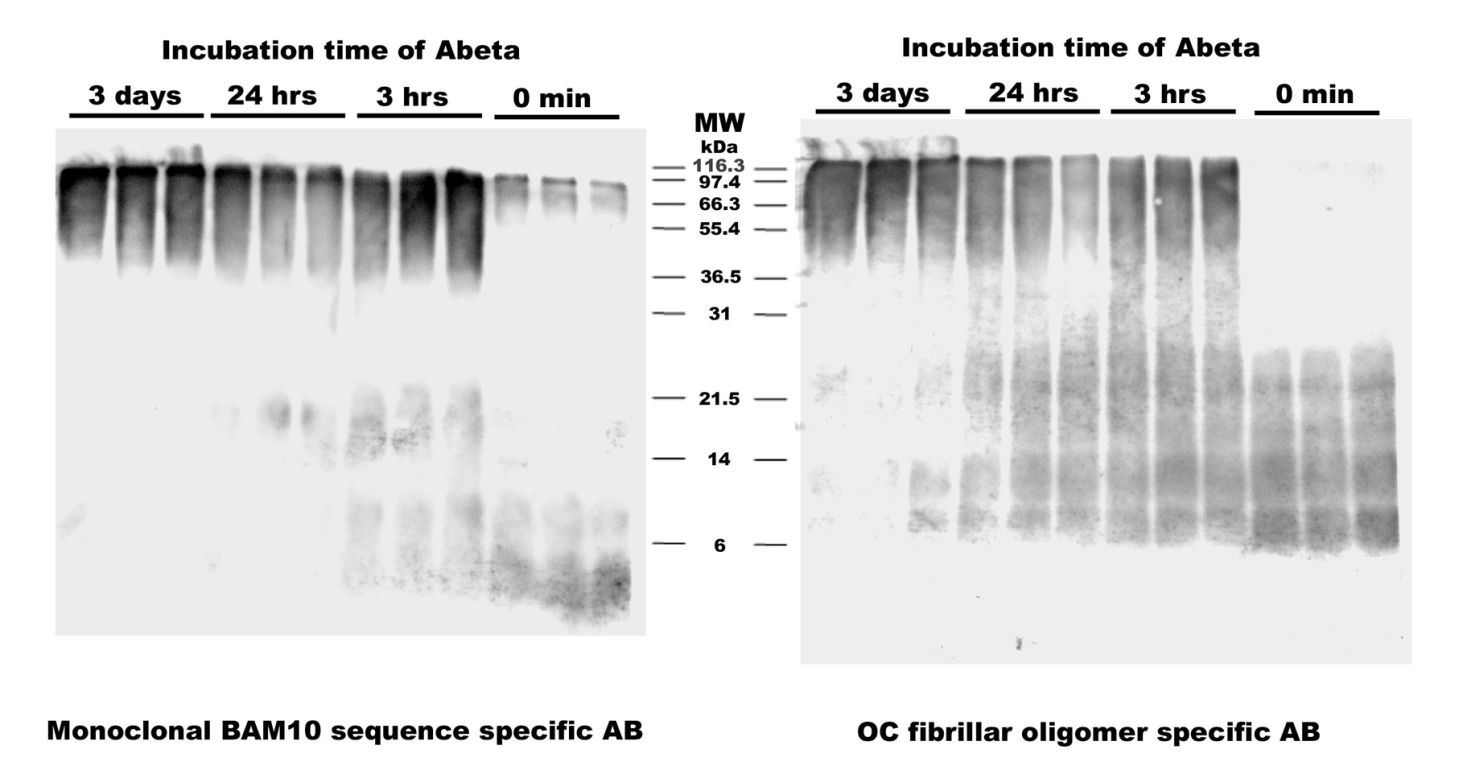

Supplement: Figure S5 — SDS-PAGE and Western Blot characterization of the Aβ oligomer sample. The results on the left (BAM10) and the right (OC) panels were obtained on identical samples. The incubation time was measured from dissolving iso-Aβ in the pH 7.4 buffer. The monomeric fraction is not stained by OC, whereas BAM10 has a limited efficiency in staining the LMW oligomers. The OC staining revealed that the monomeric population can be minimized with the incubation time and after 24 h a mixture of LMW and HMW oligomers was obtained. (TIF) [file pone.0039485.s005.tif]

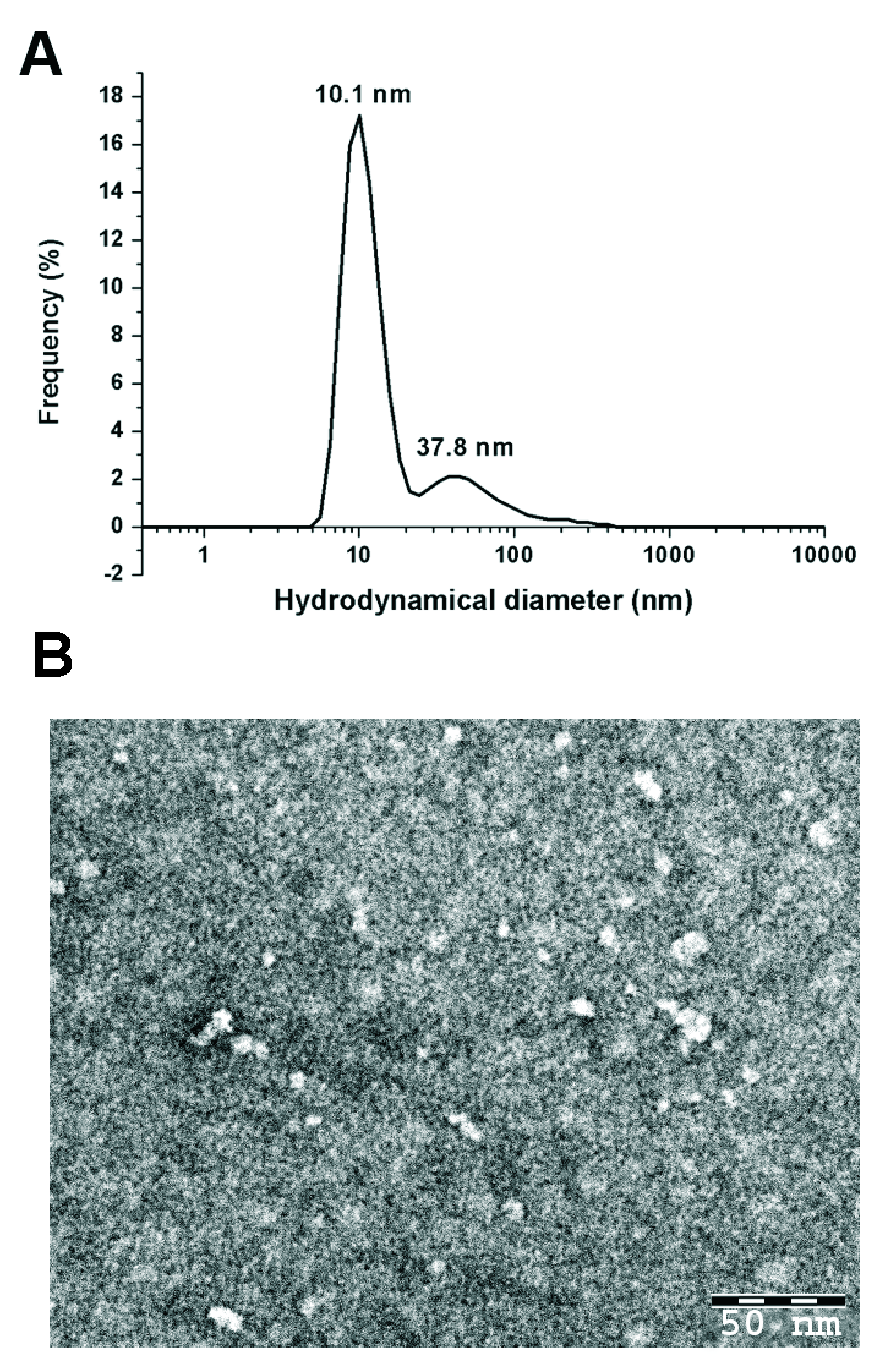

Supplement: Figure S6 — (A) DLS measurement: hydrodynamic diameter distribution of the Aβ oligomers in PBS, c = 72 μM, after incubation for 24 h. Frequencies are normalized to the intrinsic volume of the scattering particles. (B) TEM image of the oligomers on formvar-carbon coated grids, stained with uranyl acetate, visualized at 92000× magnification. (TIF) [file pone.0039485.s006.tif]

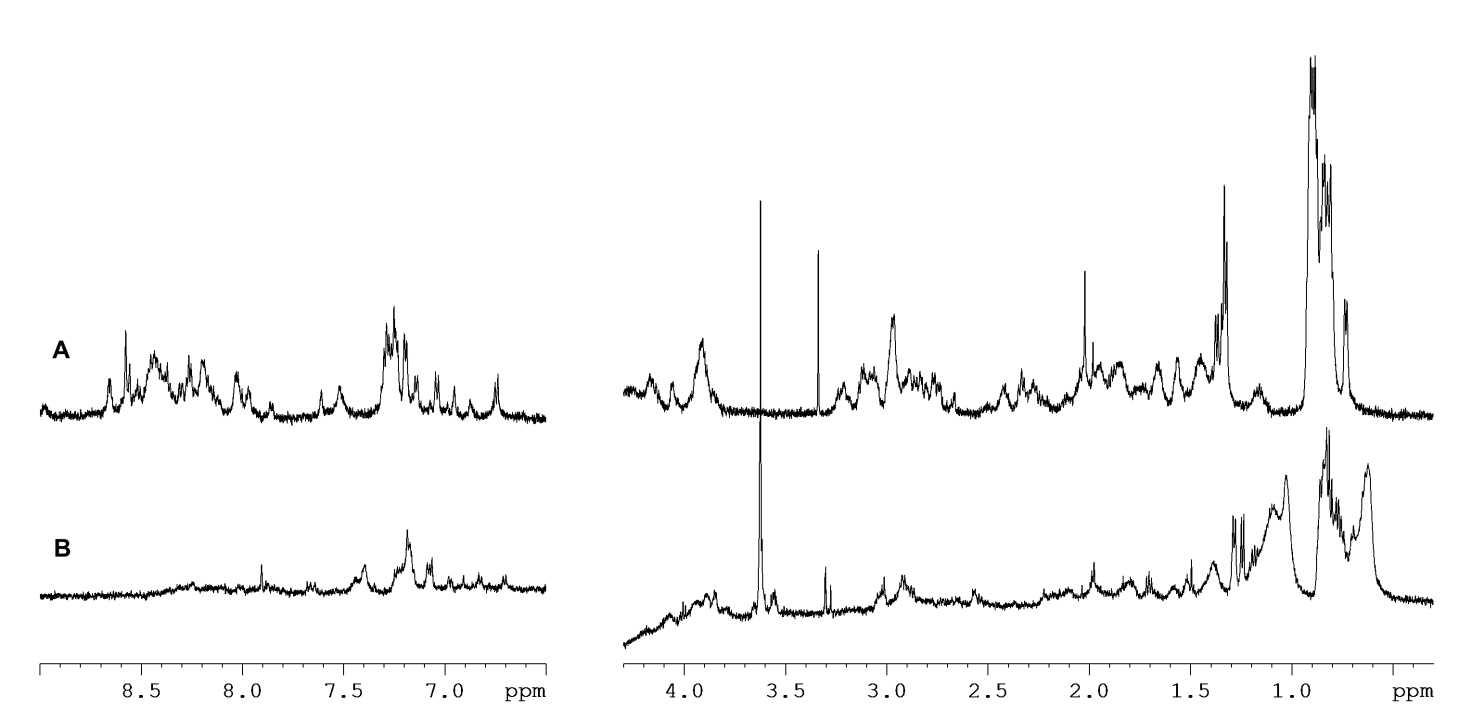

Supplement: Figure S7 — 1H-NMR spectra recorded for the Ser26 depsipeptide iso -Aβ(1–42) at pH 3 (A) and after buffering of the medium to pH 7.4 for the same sample (B). The intensities are corrected for the small dilution. (TIF) [file pone.0039485.s007.tif]

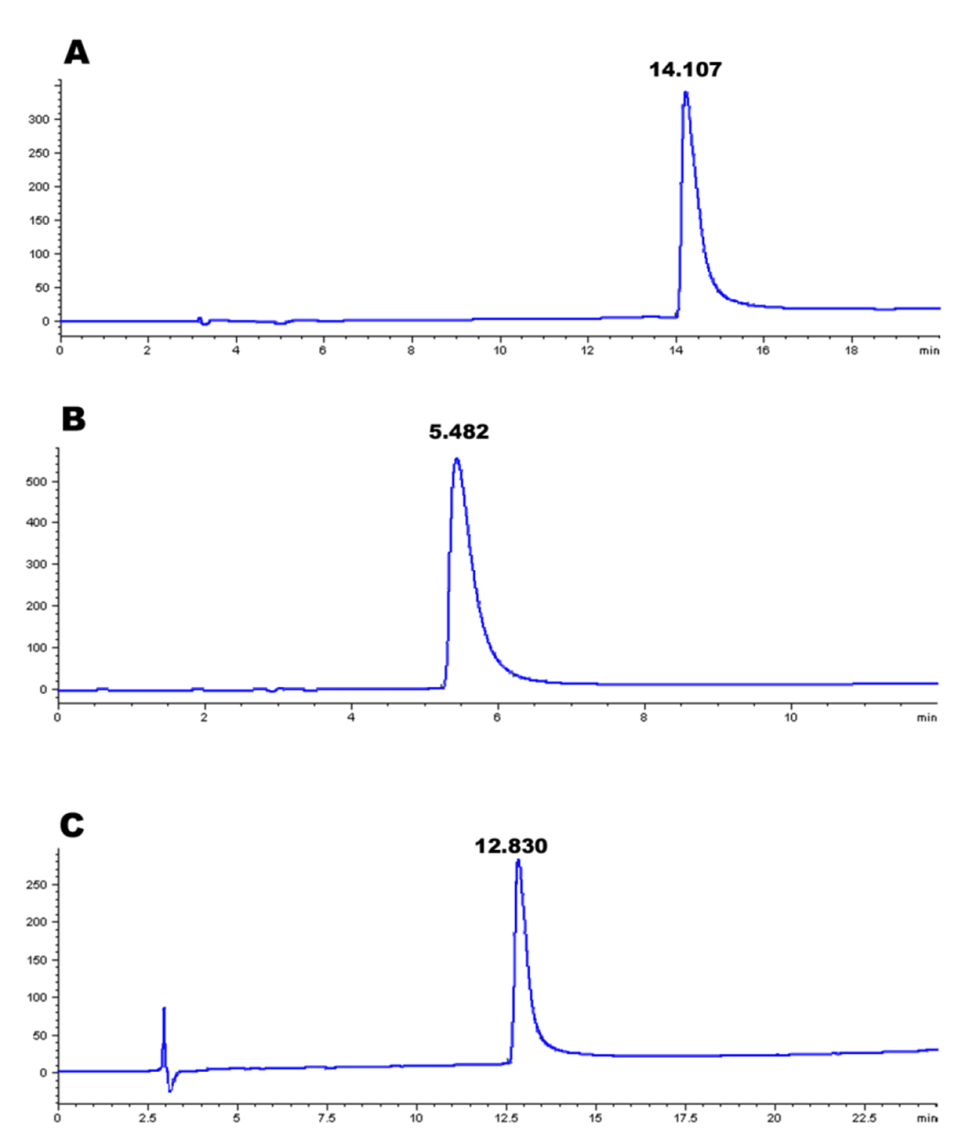

Supplement: Figure S8 — Purity and integrity of 7, 11 and biotinyl-7. Analytical HPLC chromatograms of the purified 7, 11 and biotinyl–7 are given in panels (A), (B) and (C), respectively. Conditions: solution A: 0.1% TFA in water; B: 80% ACN, 0.1% TFA in water Applied gradients: 7: 0–20% B in 20 min; 7: 40–64% B in 12 min, biotinyl-7: 25–75% B in 25 min Column Phenomenex Luna 5 C18 column 1.2 ml/min flow rate at ambient temperature. (TIF) [file pone.0039485.s008.tif]

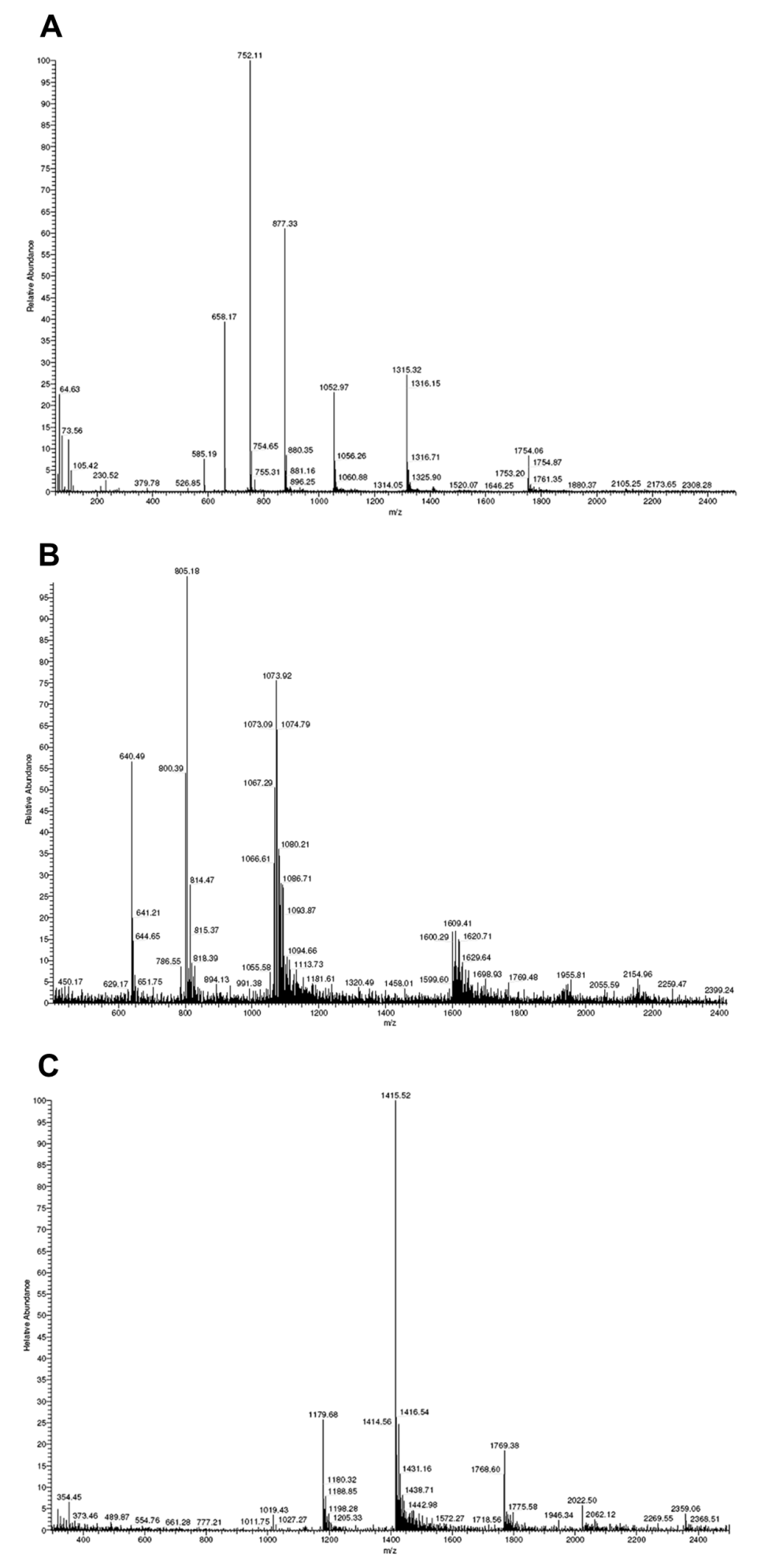

Supplement: Figure S9 — Purity and integrity of 7, 11 and biotnyl-7. ESI-MS spectra of the purified 7, 11 and biotinyl-7 are given in panels (A), (B) and (C), respectively. (TIF) [file pone.0039485.s009.tif]

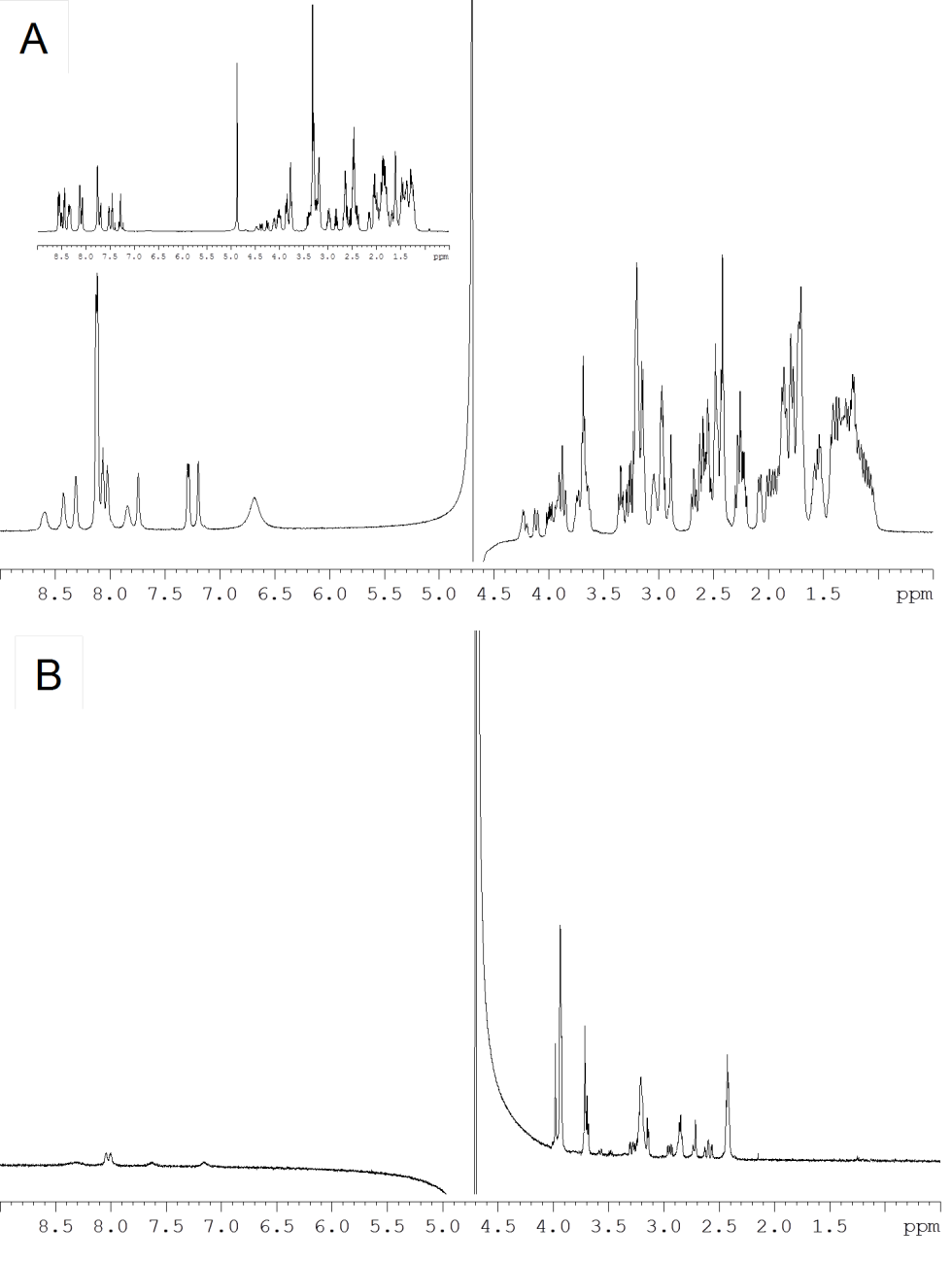

Supplement: Figure S10 — 1H-NMR WATERGATE spectra recorded in H2O∶D2O 90∶10 (buffer pH 7.4) for 7 (A) and 11 (B). The signal broadening in the amide region is due to the chemical exchange with solvent protons. Signal broadening in methanol was not observed. Inset displays spectrum of 7 in d3-MeOH. (TIF) [file pone.0039485.s010.tif]

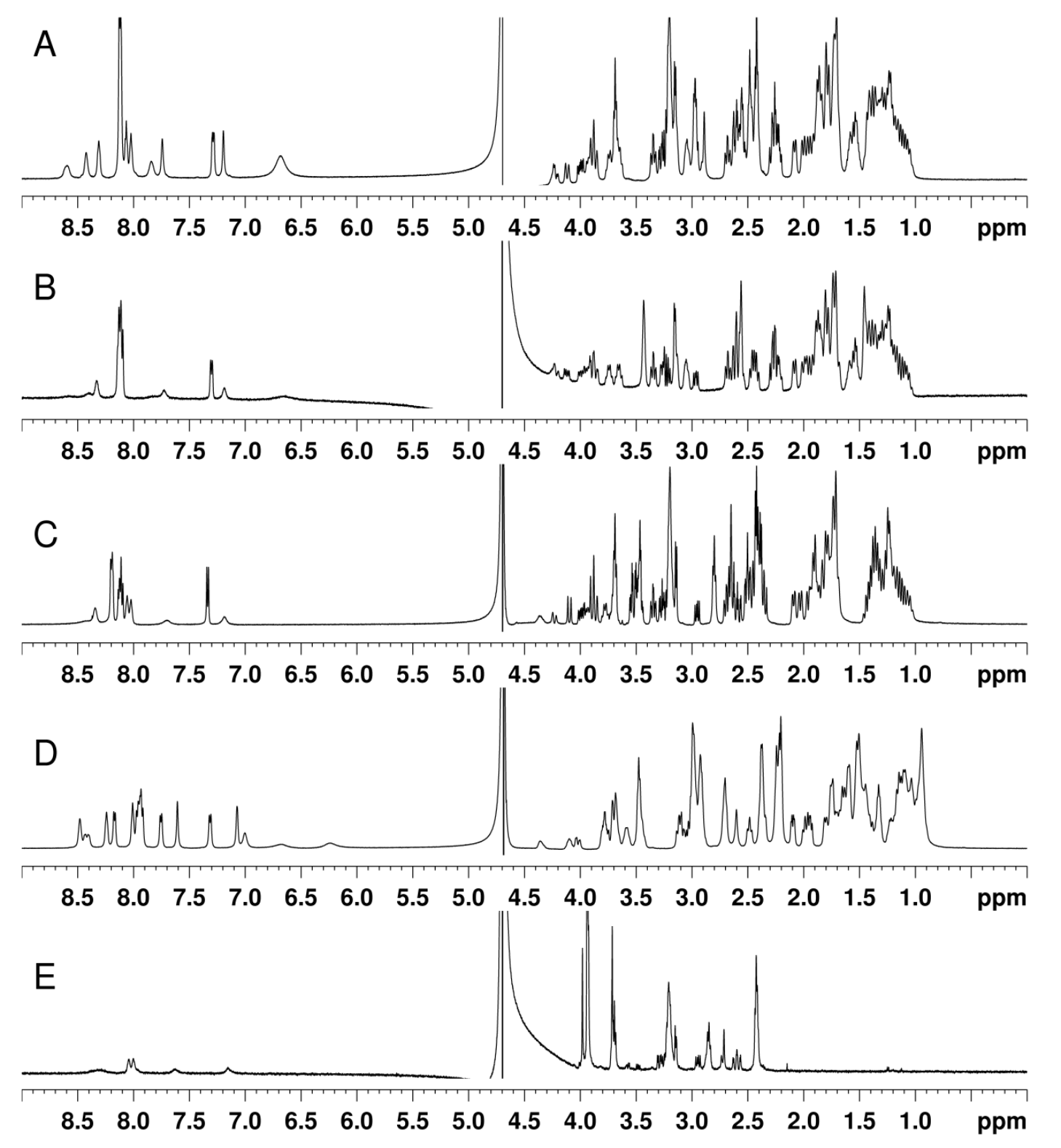

Supplement: Figure S11 — 1H-NMR WATERGATE spectra for 7–11 (A–E, respectively) recorded in H2O∶D2O 90∶10 (phosphate buffer pH 7.4). (TIF) [file pone.0039485.s011.tif]

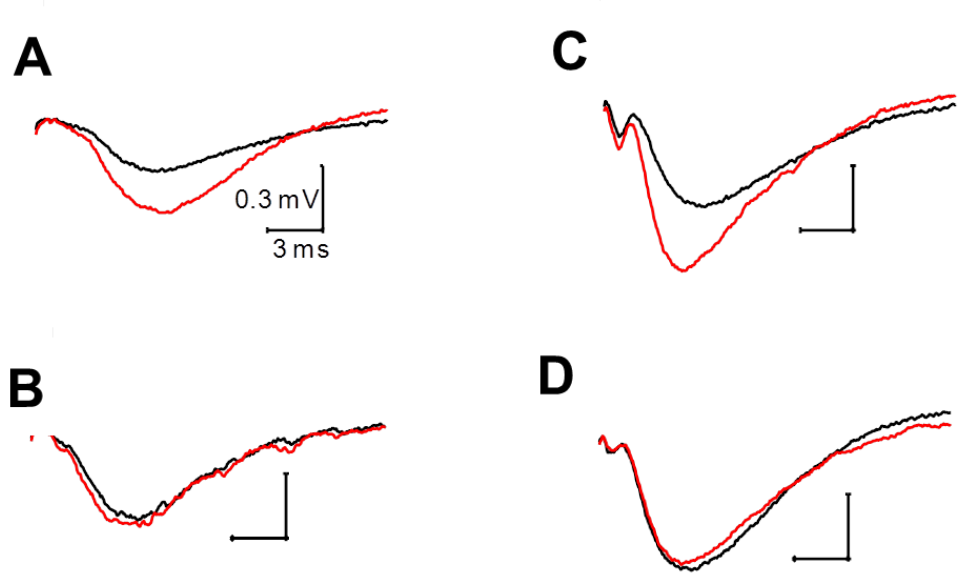

Supplement: Figure S12 — Superimposed raw data before (black) and 180 min after (red) LTP induction (A), untreated; (B), Aβ(1–42) oligomer; (C), Aβ(1–42) oligomer +7; (D), Aβ(1–42) oligomer +11). (TIF) [file pone.0039485.s012.tif]
