# Supplementary material for: A Foldamer-Dendrimer Conjugate Neutralizes Synaptotoxic β-Amyloid Oligomers
Source: PLoS One. 2012 Jul 30;7(7):e39485. doi: 10.1371/journal.pone.0039485 (PMC3408453; doi:10.1371/journal.pone.0039485)
Supplement: Table S1 — Affinities to Aβ oligomers determined for 1, 7, 8, 9 and 10 with ITC and ELISA. (DOC) [file pone.0039485.s013.doc]

**Table S1. Affinities to Aβ oligomers determined for 1, 7 and 8 with ITC and ELISA**

| **ligand** | **valency** | **ITC (K_D_)** | **ELISA (IC50)** |
| --- | --- | --- | --- |
| **7** | 4 | 6.9 ± 1.4 nM (N=0.041)^a^  281.1 ± 38.7 nM (N=0.18)^a^ | 126 nM (N = 0.25)^b^  886.2 nM (N = 0.25)^c^ |
| **8** | 2 | 721.4 ± 120.1 nM (N=0.53)^a^ | 933 nM (N = 0.5)^b^ |
| **1** | 1 | 2376.1 ± 214.4 nM (N=0.97)^a^ | 12000 nM (N = 1.0)^b^ |
| **9** | 4 | 2241.7 ± 197.7 nM (N=0.03)^a^ | 4400 nM |
| **10** | 4 | 2748.4 ± 301.4 nM (N=0.10)^a^ | 18460 nM |

a Measured at Aβ concentration of 72 µM

b Affinities were determined with stoichiometric values (N) determined with ITC

c Measured with fibrillar Aβ.
